# Host-derived chimeric peptides clear the causative bacteria and augment host innate immunity during infection: A case study of HLB in citrus and fire blight in apple

**DOI:** 10.3389/fpls.2022.929478

**Published:** 2022-12-23

**Authors:** Supratim Basu, Elena Sineva, Liza Nguyen, Narattam Sikdar, Jong Won Park, Mikhail Sinev, Madhurababu Kunta, Goutam Gupta

**Affiliations:** ^1^ New Mexico Consortium, NMC-Biolab at Santa Fe Business Incubator, Santa Fe, NM, United States; ^2^ Texas A&M Univ.-Kingsville Citrus Center, Weslaco, TX, United States

**Keywords:** plant pathogen, plant bacteria, plant–pathogen interaction, HLB (citrus greening), fire blight (*Erwinia amylovora*)

## Abstract

Bacterial diseases cause severe losses in the production and revenue of many fruit crops, including citrus and apple. Huanglongbing (HLB) in citrus and fire blight in apple are two deadly diseases without any cure. In this article, we introduce a novel therapy for HLB and fire blight by enhancing the innate immunity of the host plants. Specifically, we constructed *in silico* a library of chimeras containing two different host peptides with observed or predicted antibacterial activity. Subsequently, we performed bactericidal and toxicity tests *in vitro* to select a few non-toxic chimeras with high antibacterial activity. Finally, we conducted *ex planta* studies to show that not only do the chimeras clear the causative bacteria from citrus leaves with HLB and from apple leaves with fire blight but they also augment the host’s innate immunity during infection. This platform technology can be extended to design host-derived chimeras against multiple pathogenic bacteria that cause diseases in plants and animals of agricultural importance and in humans.

## Introduction

The citrus and apple industries provide both fresh and processed fruits to consumers for health and nourishment. However, citrus and apple growers face serious threats from chronic and emerging bacterial diseases. Effective tools are urgently needed for the treatment of bacterial diseases in citrus and apple. Of all citrus diseases, Huanglongbing (HLB) is the most devastating, which is caused by *Candidatus* Liberibacter asiaticus (*C*Las), a Gram-negative bacterium (Da Graça and Korsten, 2004; Graham et al., 2020; Hu et al., 2021) transmitted by *Diaphorina citri* Kuwayama (Asian citrus psyllids, ACP) (Alves et al., 2021). Since its first detection in 2005, the Florida citrus industry has witnessed over an 80% decline in citrus production (Singerman and Rogers, 2020). In Texas, HLB was first detected in 2012 (Kunta et al., 2012), and by 2017, it was confirmed in 28% of commercial and 40% of residential sites (Sétamou et al., 2020). In California, so far, only 3,053 isolated HLB cases have been identified and removed as mandated by the state (Kumagai et al., 2013). Although Florida, California, and Texas, the major citrus-producing states, have experienced different HLB pressures, all of them need effective, safe, and affordable tools to treat and prevent HLB. The current disease management tools consist of insecticide spray to limit the spread of psyllids (Qureshi et al., 2014; Monzo and Stansly, 2017). Moreover, nutrients, chemicals, and antibiotics have been applied for disease management (Boina and Bloomquist, 2015; Tansey et al., 2017). Unfortunately, none of these disease management tools provide a cure for HLB. Fire blight, like HLB, is a destructive disease of apples and pears. Fire blight is caused by the bacterium *Erwinia amylovora*, which infects blossoms, fruits, vegetative shoots, woody tissues, and rootstock crowns (Johnson and Stockwell, 1998). It is estimated that US apple producers suffer an average annual loss of US $100 million due to fire blight (Kharadi et al., 2021). The diversity of host tissues susceptible to infection, combined with the limited number of available and effective disease management tools, has made it difficult to stop or slow the progress of fire blight epidemics.

Over a decade ago, we introduced the concept of host-based therapy for the treatment and prevention of bacterial diseases in humans and plants (Lehnert et al., 2001; Hong-Geller et al., 2004; Vuyisich et al., 2008). The application of this concept was successful in combating diseases caused by intact bacteria or the toxins secreted by them. Subsequently, for the application of host-based therapy, we focused primarily on enhancing the innate immunity of humans and plants, the first line of defense against invading pathogens (Kunkel et al., 2007). It should be noted that a plant’s innate immune repertoire contains pathogenesis-related (PR) or defense peptides/proteins to clear pathogens or block pathogenesis (Ali et al., 2018). However, the evolution of bacterial resistance often suppresses the action of the PR proteins in host defense (Jones and Dangl, 2006). Therefore, we developed strategies to introduce sequence/structure modifications in the PR peptides/proteins to overcome bacterial resistance while retaining high activity against invading pathogens.

One of the successful applications of our strategy involved the design of helix-turn-helix (HTH) peptides and their use in the treatment of two bacterial diseases, namely, Pierce’s disease (PD) in grape caused by xylem-limited *Xylella fastidiosa* (Xf) and HLB in citrus caused by phloem-limited *C*Las (Gupta and Stover, 2020). The HTH peptides were designed by joining two identical helical amphipathic peptides with a sharp type II GPGR turn (Sharma et al., 2014; Gupta and Stover, 2020). While each helix had homologous segments in grape and citrus proteins, the whole length of the artificially constructed HTH peptides showed very little homology with any grape or citrus protein segment. Therefore, we performed toxicity assays to verify that the HTH peptides are not toxic to plant leaves or human cells at the active dose (Gupta and Stover, 2020). In field trials, two HTH peptides (peptide names: 28P-2 and 36P-1) showed efficacy for the treatment of PD in grapevines and HLB in citrus (Gupta and Stover, 2020). Laboratory studies revealed that 36P-1 was 14 times more active on *C*Las than 28P-2 (Gupta and Stover, 2020). However, 36P-1 appeared toxic at the treatment dose, which required appropriate sequence/structure modifications or, better yet, the development of a general strategy for the design of a new class of peptides with similar activity but with no toxicity.

In this article, we introduce a general strategy in which two different peptide segments, instead of two identical ones, were combined. Again, these peptide segments were selected from the proteins belonging to the plant’s innate immune repertoire (Cunsolo et al., 2020; Huan et al., 2020; Li et al., 2021). The goal was to demonstrate that the combination of two different peptides results in a chimera suitable for the treatment of bacterial diseases in plants. In addition, we wanted to show that these chimeras not only show bactericidal activity on infected plants but also augment a plant’s innate immune system during infection. These chimeric peptides were constructed by joining two different segments from citrus/apple proteins. These segments in isolation show (or are predicted to show) the membranolytic/killing activity of Gram-negative bacteria. Typically, the individual segments show low bactericidal activity; however, when present in a chimera scaffold, high activity is observed due to their synergy. These segments are generally unstructured in isolation but can form α/β structures when they encounter hydrophobic solvents akin to the bacterial membrane (Huynh et al., 2021). However, the α/β chimera scaffold may facilitate the formation of alpha or beta structures even without the association of a bacterial membrane. The following steps led to the identification of non-toxic α/β-peptide chimeras that clear bacteria and augment the innate immunity during infection. Firstly, we performed molecular modeling to construct a library of peptide chimeras by joining two antibacterial α/β segments from citrus/apple proteins and selected energetically stable ones with high predicted activity. Secondly, we custom synthesized the selected chimeras and measured the minimum inhibitory concentration (MIC) against Gram-negative *Escherichia coli*. Thirdly, we determined the toxicity of the chimeras that showed high activity on plant and human cells. Fourthly, the chimeras that were non-toxic and had high activity were examined in an *ex planta* detached leaf assay for anti-*C*Las and anti-*amylovora* activity. Finally, we determined the expression of selected genes to show that the chimeric peptides augmented the innate immunity in citrus/apple during infection.

## Results

### Identification of chimeric peptides with *in vitro* bactericidal activities on *E. coli*

Antibacterial peptides, which are present in many plant proteins, can either be linear or disulfide (S–S) bridged (Li et al., 2021). The linear peptides were the main focus of the current study. As shown in **Table 1**, four types of linear antibacterial peptides were considered (Huan et al., 2020): i) amphipathic peptides such as KKLIKKILKIL/KKLFKKILKYL, designated as unit A; ii) FWQ-containing basic peptides, such as FWQRRIRRWRR/FQWQRNIRKVR, designated as unit C/B; iii) R/W-rich peptides, such as RRWWRWWR, designated as unit D; and iv) IERSTNLDWYKGPTLL or unit E identified from plant extracts (Cunsolo et al., 2020). A library of chimeras was constructed by joining two different units with a linker containing one to eight amino acids. The initial structure of each chimera was obtained using homology modeling (Waterhouse et al., 2018) and energy minimized in a vacuum using the GROMOS96 (CHARMM: the biomolecular simulation program 2009) force field (van Gunsteren et al., 2006). Previously, we have performed molecular dynamics (MD) simulation of various peptides in a water/(lipid bilayer) system to visualize the three determinants of the antibacterial activity of helical amphipathic peptides (Gupta and Stover, 2020; Huynh et al., 2021), namely, membrane attachment, insertion, and rupture. The MD simulations also revealed that the antibacterial activity depended on the helical content and stability of the chimera, the total charge, and the relative disposition of the charged *vs*. hydrophobic amino acids on the surface or in the interior of the structure. These parameters in the energy-minimized structures of the chimeras allowed their empirical ranking in terms of antibacterial activity. The top-ranked chimeric peptides were custom synthesized in milligram quantities. We then measured the MICs of the peptides that killed all 5 × 10^5^ colony-forming units (cfu) of a bacterial culture after 24 h of incubation (Wiegand et al., 2008). The MIC assay was performed on a 96-well plate by serial dilution of the peptide in the concentration range 0.02–20 μM. The growth of the *E. coli* strain BL21 (and, in some cases, ATCC25922) was monitored by measuring the optical density (OD)/cfu. This produced the range of MICs, above which no bacterial growth was observed. **Table 1** shows the MIC ranges of the individual units A–E and the chimeras constructed by joining two of them. The chimeric peptides with MICs above 20 μM were not further studied. **Supplementary Tables SIA**, **SIB** show various fragments of the citrus and apple proteins that are homologous to the various chimeras shown in **Table 1**, which shows segments homologous to: i) single units on the N/C-terminal of the chimeras; ii) linkers joining them; and iii) the chimera fragments containing the N-terminal, the linker, and the C-terminal.

**Table 1:** Minimum inhibitory concentrations (MICs, in micromolars) of the single-unit antibacterial peptides and the chimeras constructed with the single antibacterial units.

Code	Type of scaffold	Amino acid sequence	MIC (mM)
11P-1	Unit A	KKLIKKILKIL (KKLFKKILKYL)^a^	10–20
11P-2	Unit B	FQWQRNIRKVR^b^	>20
11P-3	Unit C	FWQRRIRRWRR	>20
8P-1	Unit D	RRWWRWWR	>20
16P-1	Unit E	IERSTNLDWYKGPTLL	>20
Gen-1	Chimera AA-1	KKLPKEILKILGSGYGSLPKEILKILELKK	5-10
Gen-2	Chimera AC-7	KKLPEKILEILGSGYKKLPFWQRRIRRWRR	2.5–5
30P-1	Chimera AC-1	KKLIKKILKILGSGYGSPGFWQRRIRRWRR	1.25–2.5
30P-2	Chimera CA-1	FWQRRIRRWRRGSGYGSPGKKLIKKILKIL	1.25–2.5
30P-3	Chimera AC-2	KKLPKKILKILGSGYGSPGFWQRRIRRWRR	0.6–1.25
27P-1	Chimera DC-1	RRWWRWWRGSGYGSPGFWQRRIRRWRR	>20
27P-2	Chimera CD-1	FWQRRIRRWRRGSGYGSPGRRWWRWWR	5–10
27P-3	Chimera AC-3	RKPARKVLKILGRGSEFWQKRVRRWRR	10–20
UGK-1	Chimera CD-2	RLPKAFQWQRRLRRWRRPYSPGRRWWRWWR	5–10
UGK-5	Chimera CD-3	RLPEAFQWQRRLRRWRRPDSPGRRWWRWWR	5–10
UGK-9	Chimera CD-4	RLPEAFQWQRNIRKVRRPDSPGRRWWRWWR	5-10
UGK-13	Chimera AC-3	KKLPEKILKILESGYGSPGFWQRRIRRWRR	0.625–1.25
UGK-17	Chimera AC-4	KKLPEKILKILESLKGSPGFWQRRIRRWRR	1.25–2.5
UGK-21	Chimera AC-5	KKLPQKLLEILKSLKGSPGFWQRRIRRWRR	1.25–2.5
UGK-25	Chimera AC-6	KKLPEKLLEILKSLEGSPGFWQRRIRRWRR	2.5–5
UGK-29	Chimera AD-1	KPRGSEQLQELTRRLLDSPLERRWWEWMRR	>20
UGK-33	Chimera AD-2	KPRLSEQLQELTRRLLDSPLERRWWEWWRR	>20
UGK-37	Chimera AD-3	KPRGSEQLQELTRRLLDSPLERRFWQWMRR	>20
UGK-41	Chimera AD-4^c^	KRPEELLQKLKSLEGSKAHLQHHDWTSK	>20
UGK-45	Chimera AD-5^c^	LPKRLEELLQKLKSLEGSKAHEKLHDWTRK	>20
UGK-49	Chimera AD-6^c^	QPKRLEELLEKLKSLEGSKAHEKLHDWTRK	>20
UGI-7	Chimera DC-2	RRWWRWWRGSYGSVYGSPGFWQRRIRRWRR	5–10
I27AB	Chimera E+1	IERSRNLDWYKGPTLLDALKNLNEGKR	>20
I31LB	Chimera E+2	IERSRNLDWYKGPTLLDALKNLNEGKRPSDK	>20
L19GA	S–S bridged#2	KC2RRLC6YKQRC11VTYC15RGRQ^d^	1.25–2.5

a Homolog of A: a hybrid of cecropin and melittin.

b Very similar to C: intrachain disulfide (S–S) bridges C2–C15 and C6–C11.

c Distant homologs of AD.

d S–S bridged.

**Figure 1:**
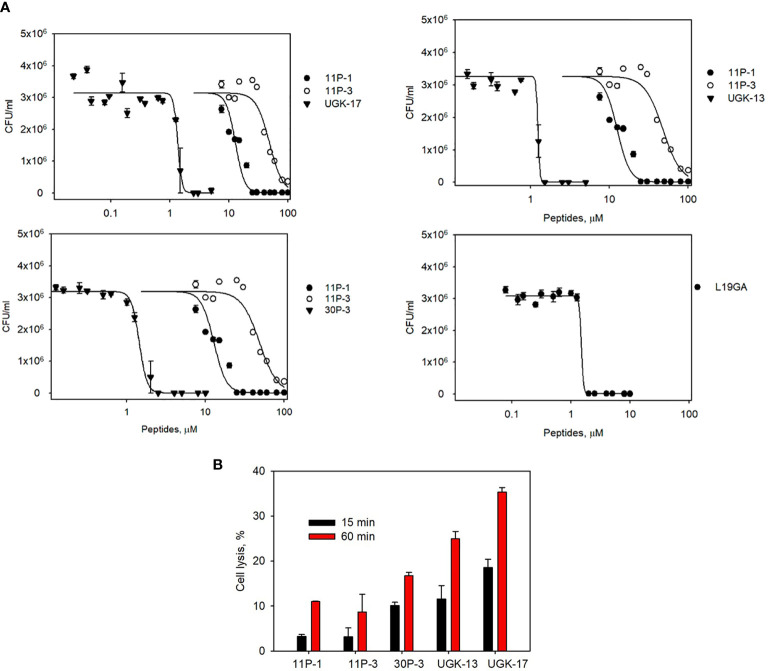
**(A)** Bioluminescence data of the viability of *Escherichia coli* BL21 at different peptide doses for the α/β chimeras UGK-13, UGK-17, and 30P-3. Also shown are the dose–response curves for the single-unit constituents of the chimeras, i.e., 11P-1 and 11P-3, and the dose–response curves for L19GA with two β-strands stabilized by two S–S bridges. *E coli* BL21 cells (5 × 10^5^ cfu)were incubated with peptides for 24 h. **(B)** Bacterial cell lysis by the different peptides was measured using the luciferase assay. Bacterial cell death was observed by the lysis of *E coli* BL21 (pACYC184-nLuc) cells upon incubation with corresponding antibacterial peptides by measuring the luciferase activity in the cell-free supernatants at 15 min and 1 h post-treatment.

The chimeric peptides with MICs in the range 0.6–2.5 μM were considered potential bactericidal agents and were selected for the bioluminescence assay for determining the exact MIC values. The bioluminescence assay (Paciello et al., 2013) helps measure the number of viable bacterial cells in a culture based on the quantitation of the ATP present. It should be noted that ATP is present in live (metabolically active) cells, but not in dead cells. **Table 2** lists the bactericidal activities of the promising antibacterial chimeras on *E. coli* BL21. Bactericidal activity was expressed in terms of IC_50_ and IC_99_–MIC. **Table 2** also includes the IC_50_ and IC_99_–MIC of the single units A–E and those of the equimolar mixtures of A (11P-1) and C (11P-3). Chimeras AC and CD were the most promising antibacterial agents. The synergistic effects of units A and C in chimeras should also be noted, as evidenced by the much lower MICs of these chimeras compared to those of the individual A/C units, or the equimolar mixtures of units A and C (see **Supplementary Figure S2** for additional explanations). **Figure 1A** shows the cfu per milliliter values from the bioluminescence assay at different peptide concentrations, which reveals the slope of the transition from live to dead bacteria upon peptide binding to bacteria, and Hill’s coefficient, an index of cooperative binding (Paciello et al., 2013). We also measured bacterial cell lysis by the different peptides using the NanoLuc luciferase assay. For this assay, *E. coli* BL21 (pACYC184-nLuc) was treated with 10 µM peptides and the luciferase activity was monitored in the supernatant at 15 min and at 1 h post-treatment. Peptide concentrations (higher than the MICs) were needed to observe the early effect on *E. coli*. The measured luciferase activity is proportional to the number of lysed bacterial cells. **Figure 1B** shows the percentage of bacterial lysis by the single units (11P-1 and 11P-3) and their α/β chimeras (30P-3, UGK-13, and UGK-17). It appears from the data shown in **Figures 1A, B** that UGK-17 was the most active chimera on *E. coli*. The synergy of the antibacterial activities of 11P-1 and 11P-3 in the chimera 30P-3 is illustrated in **Supplementary Figure S1**, i.e., the activity of the chimera was higher than the additive effects of 11P-1 and 11P-3 (in fact, there was a negative interference of the two when added together in equimolar concentrations).

**Table 2:** Bactericidal activity of the selected α/β peptides measured using the bioluminescence assay.

Peptide	IC_50_ (µM)	IC_99_ (µM) (~MIC)	Hill’s coefficient
UGK-13/chimera AC-3	1.24 ± 0.02	1.40 ± 0.03	36
UGK-17/chimera AC-4	1.36 ± 0.04	1.97 ± 0.06	12
30P-3/chimera AC-2	1.45 ± 0.09	2.41 ± 0.11	9
Gen-1/chimera AA-1	10.22 ± 0.07	11.4 ± 0.07	41
Gen-2/chimera AC-7	2.83 ± 0.14	4.48 ± 0.22	10
UGK-19GA/S–S bridged#2	1.49 ± 0.22	1.88 ± 0.29	20
11P-1/unit A	12.94 ± 0.62	29.38 ± 0.75	6
11P-3/unit C	48.98 ± 2.79	160.80 ± 3.25	4
11P-1+11P-3/unit A + unit C	40.97 ± 1.11	60.00 ± 1.63	12

The IC_50_ and IC_99_ (which are the MICs) and Hill’s coefficients were calculated from the dose–response (bioluminescence vs. peptide concentration) sigmoidal curve.

**Figure 2:**
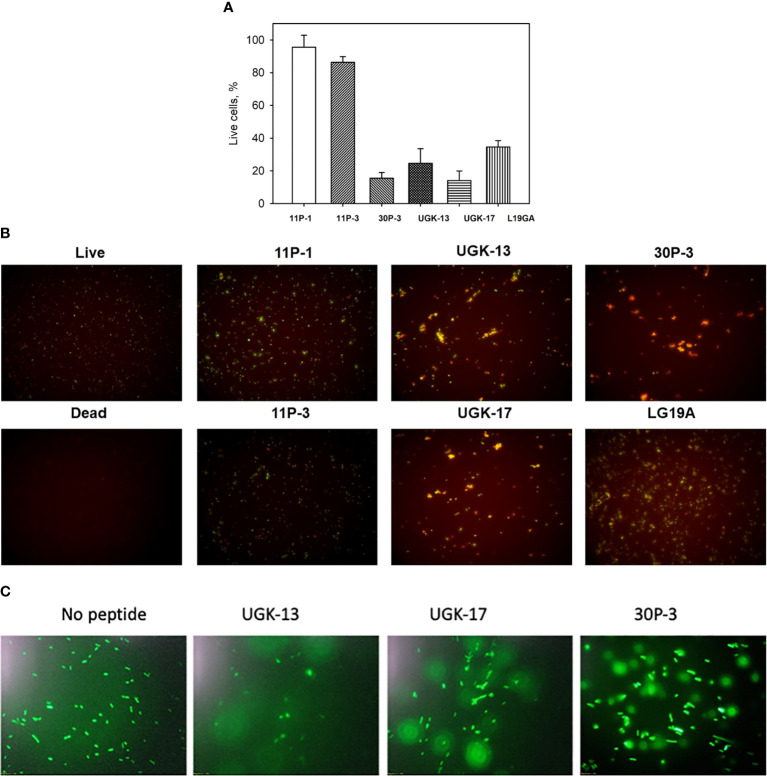
Two-color fluorescence assay after 1 h of peptide incubation of *Eschrichia coli* BL21 cells (5 × 10^5^ cfu). **(A)** Percentage of live cells by treatment with different peptides at 20 μM concentration. **(B)** Fluorescent images of live and dead bacteria for the same experiment as **(A)**. **(C)** Single-color fluorescence images of green fluorescent protein (GFP)-labeled BL21 after 20 μM peptide treatment for 24 h.

The ratio of live/dead *E. coli* BL21 cells due to peptide treatment was also monitored using a fluorescent-based assay (Chen et al., 2021). For this assay, two fluorescent dyes, SYTO9 and propidium iodide (PI), were used. SYTO9 stains green in both live and dead cells, whereas PI only intercalates and stains red the DNA of the dead cells or the cells with ruptured membranes. **Figure 2A** shows the percentage of live cells after 1 h of 20 μM peptide treatment on 5 × 10^5^ cfu of *E. coli* BL21 relative to the live and dead cell controls, whereas **Figure 2B** shows the same data presented visually by fluorescent images of green- and red-labeled BL21 upon peptide treatment. Peptide concentrations (higher than the MICs) were needed to observe finite changes in fluorescence in treated and untreated *E. coli*. The single units (11P-1 and 11P-3) and their α/β chimeras (30P-3, UGK-13, and UGK-17) are shown in **Figures 2A, B**. In addition, the effect of L19GA on *E. coli* BL21 is shown in **Figures 2A, B**. It should be noted that the L19GA peptide contains two β-strands with two intrachain disulfide bridges. Finally, we performed single-color fluorescent microscopy. For this, the peptides were incubated for 1 h with *E. coli* BL21 (pACYC184-GFP). **Figure 2C** shows the fluorescent micrographs after 1 h of peptide treatment. The peptide that shows the highest clearance or the lowest reduction in green fluorescence is the most active on *E. coli*. Thus, the single- and two-color fluorescent assays showed that UGK-17 was the most active peptide on *E. coli*.

### Plant and human cell toxicity of the peptide chimeras with *in vitro* bactericidal activity on *E. coli*

**Figure 3:**
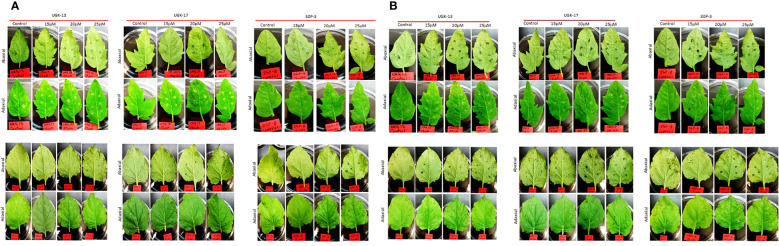
Toxicity monitoring after infiltration with different peptides at 15–25 μM concentration for 24 and 96 h. **(A)** Tomato leaves (*top*) and tobacco leaves (*bottom*) for 24 h. **(B)** Tomato leaves (*top*) and tobacco leaves (*bottom*) for 96 h.

Both plant and human toxicity analyses were performed on the chimeras UGK-13, UGK-17, UGK-9, and 30P-3. Plant toxicity was measured by infiltrating 1 ml of 15–25 μM peptide solution to each tomato and tobacco leaf using a 1-ml syringe (Inui Kishi et al., 2018). The peptide concentrations were 10 times or higher than their MICs on *E. coli*. Each leaf was abaxially and adaxially infiltrated at four to six spots. The infiltrated plants were kept for 96 h in a growth chamber. The infiltrated leaves were visually analyzed at 24, 48, 72, and 96 h. When a peptide is toxic at a given concentration, the corrosive effect spreads in the leaf beyond the infiltration spots. **Table 3** summarizes the leaf infiltration data, which show that the chimeras (UGK-13, UGK-17, UGK-9, and 30P-3) were not toxic to tomato and tobacco at concentrations of 15–25 μM even after 96 h of leaf infiltration. **Figures 3A, B** show the tomato and tobacco leaves after 24 and 96 h of 15–25 μM peptide infiltration using UGK-13, UGK-17, and 30P-3 relative to the water-infiltrated control. **Supplementary Figure S2** displays the data on all four peptides (UGK-13, UGK-17, UGK-9, and 30P-3) for all 24, 48, 72, and 96 h of infiltration. Similar to the control, the peptide-infiltrated tomato and tobacco leaves showed no corrosion beyond the infiltrated spots.

**Table 3:** Toxicity of the selected chimeras on tobacco and tomato leaves by infiltration of 15–25 mM peptide solution and the corrosive effect beyond the infiltration spots after 24–96 h.

Peptides	Control	Dose 1 (mM)	Dose 2 (mM)	Dose 3 (mM)	Volume infiltrated (ml)	Toxicity (monitored up to 96 h)
UGK-13	Water	15	20	25	20	Not toxic
UGK-17	Water	15	20	25	20	Not toxic
30P-3	Water	15	20	25	20	Not toxic
UGK-9	Water	15	20	25	20	Not toxic

Water was used as the non-toxic control.

**Figure 4:**
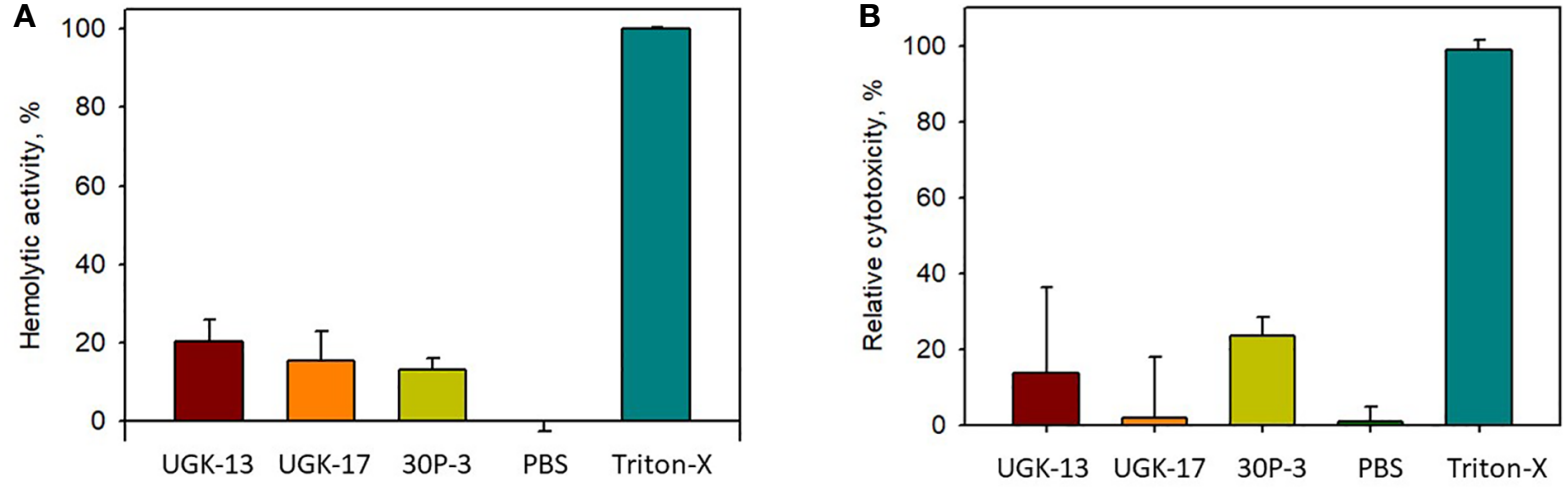
Cell viability from treatment with different peptides at 20 μM concentration with the hemolytic **(A)** and MTT **(B)** assays.

The toxicity of the chimeric peptides was also measured on human cells using human erythrocytes and human embryonic kidney (HEK) cells. Hemoglobin release *via* hemolysis or membrane rupture of erythrocytes provides a rapid measure of toxicity of the chimeric peptides (Sineva et al., 2009). **Figure 4A** shows the percentage of hemolysis of human erythrocytes by UGK-13, UGK-17, and 30P-3 at 20 μM relative to phosphate-buffered saline (PBS; negative control) and 0.01% Triton X-100 (positive control). The cytotoxicity of the HEK cells due to the three chimeric peptides was measured with the MTT [3-(4,5-dimethylthiazol-2-yl)-2,5-diphenyltetrazolium bromide] assay (Denizot and Lang, 1986), which assessed the metabolic activity of live cells by monitoring the level of intracellular NAD(P)H-dependent oxidoreductase enzymes. **Figure 4B** shows the percentage of cytotoxicity of the HEK cells treated with 20 μM UGK-13, UGK-17, and 30P-3 relative to PBS and Triton-X. The three chimeras at 20 μM concentration (10 times higher than their MICs) showed low (<20%) toxicity to human erythrocytes and HEK cells.

### Structural models of the chimeras with bactericidal activity and low toxicity

**Figure 5:**
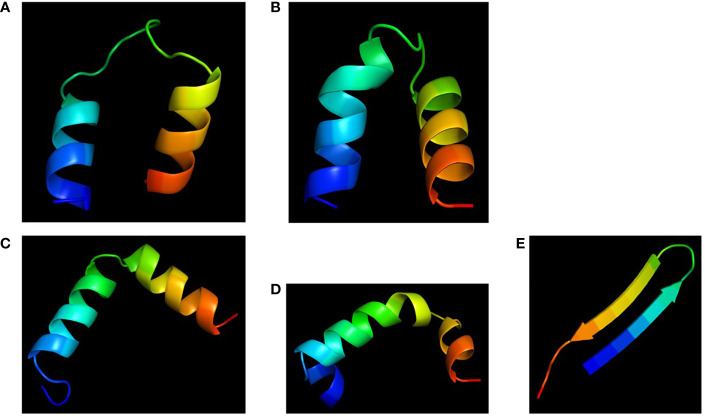
Cartoon diagrams (*blue* to *red* from N- to C-terminal) of the different peptides. **(A)** 30P-3, KKLPKKILKILGSGYGSPGFWQRRIRRWRR. **(B)** UGK-13, KKLPEKILKILESGYGSPGFWQRRIRRWRR. **(C)** UGK-17, KKLPEKILKILESLKGSPGFWQRRIRRWRR. **(D)** UGK-9, RLPEAFQWQRNIRKVRRPDSPGRRWWRWWR. **(E)** L19GA, KC2RRLC6YKQRC11VTYC15RGRQ (S–S bridges: C2-C15 and C6-C11).

Molecular models of the four chimeras (30P-3, UGK-13, UGK-17, and UGK-9) are shown in **Figures 5A–D**. As abovementioned, the models were energy minimized after obtaining the initial homology-based structures. It should be noted that 30P-3, UGK-13, and UGK-17 belong to the AC chimera family, whereas UGK-9 belongs to the CD chimera family (**Table 1**). The three AC chimeras contain α-helical units of A and C. In all three AC chimeras, the fourth residue “I” in unit A was replaced by “P” to better nucleate the N-terminal α-helix. The linkers are different in the three AC chimeras in terms of sequence and length. The linkers also adopt different conformations, judging by the peptide backbone angles (*ϕ* and *ψ*) in the energy-minimized models. The linker in 30P-3 (GSGYGSPG) adopts an extended (or β) conformation, whereas the linker in UGK-13 (GYG) forms a sharp GY turn followed by a short β-strand connecting the C-terminal helix. Finally, the single amino acid S linker in UGK-17 forms a β-strand (or a kink) between the N- and C-terminal helices. UGK-9, a member of the CD chimera family, forms three α-helices. The N-terminal helix is connected by a single amino acid β-stranded “F” to the central helix, which is connected to the C-terminal helix by an SPGR turn. In the CD chimera UGK-9, a segment of RLPEA is added to unit C to extend the N-terminal α-helix. L19GA is a single peptide fragment with antibacterial activity on *E. coli* BL21 (see **Table 1**) with homologs in citrus and apple (see **Supplementary Tables SIA, SIB**). Two β-strands are joined by a YKQR turn. Thus, in the study, we considered peptides with α-helices and β-strands (designated as α/β peptides). The individual α-helix or β-strand may contribute to the antibacterial activity. In addition, a β-strand may also define the conformation of a linker in the chimera. One important feature of the α/β peptides is the relative dispositions of the basic, acidic, and hydrophobic amino acids, which are important for the stability and activity of the peptides (see **Supplementary Figure S3** for further details).

### *Ex planta* antibacterial activities of selected chimeric peptides against *C*Las and *E. amylovora*

**Figure 6:**
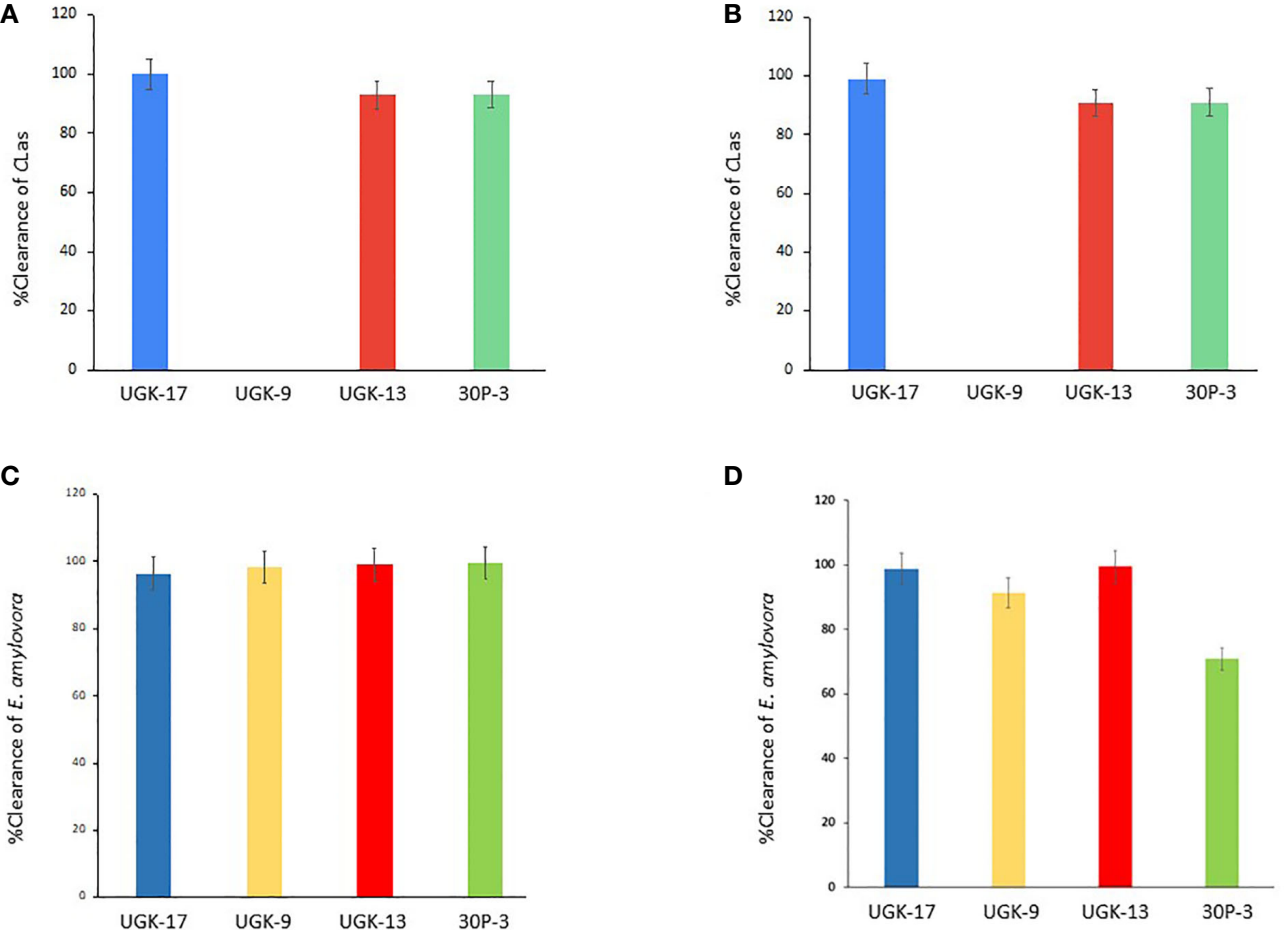
Bacterial clearance of *Candidatus* Liberibacter asiaticus (*C*Las) **(A, B)** and *Erwinia amylovora* **(C, D)** by DNA and RNA quantitative PCR (qPCR).

After determining the antibacterial activities of 30P-3, UGK-13, UGK-17, and UGK-9 and their lack of toxicity to plant leaves and human cells, we examined whether these chimeras clear the causative bacteria from infected citrus and apple leaves collected from the field. We collected the citrus grapefruit leaves with HLB from Texas and the Red Delicious apples with fire blight from New Mexico. *Ex planta* bactericidal assays are particularly relevant since *C*Las cannot be cultured in the laboratory. The *ex planta* assay allowed comparison of the clearance of *C*Las and *E. amylovora* by various chimeras under the same condition. The *ex planta* assay involved the collection of infected citrus and apple leaves and the measurement of the bacterial load by quantitative PCR (qPCR) in peptide-treated and untreated leaves. Specifically, the infected leaves (pretested with or without symptoms) were collected from the field. The petioles of the leaves were dipped into sealed Eppendorf tubes containing 1–2 ml of 20–25 μM peptide solution or water (untreated control). It was estimated that the absorption of 0.8–1 ml of peptide solution was needed for complete clearance. It took about a day for the apple leaves while it took up to 3 days for the citrus leaves to absorb 0.8–1 ml of peptide solution. The treated and untreated leaves were then crushed and the total DNA/RNA was extracted. The bacterial load was measured from the extracted DNA/RNA using qPCR with primers specific to the 16S RNA locus in *C*Las (Bao et al., 2020) and a DNA locus in the plasmid pEA29 in *E. amylovora* (Santander et al., 2019). It should be noted that both live and dead bacterial loads are measured by qPCR of DNA, whereas only the live bacteria are counted by qPCR of RNA. Moreover, primers specific to citrus and apple reference genes were used to ensure that the amplification of these genes was unaltered by treatment, thereby confirming that the peptides were specific to the bacteria. The cytochrome oxidase (*COX*) of citrus and the ubiquitin of apple were chosen as the reference genes for the qPCR of DNA, while the glyceraldehyde-3-phosphate dehydrogenase (*GAPDH*) gene in citrus and apple was selected as the reference gene for the qPCR of RNA. **Table 4** shows the effect of 25 μM 30P-3, UGK-9, UGK-13, and UGK-17 on the *C*_t_ values and the cfu of *C*Las and *E. amylovora* by DNA and RNA qPCR. Typically, treatment showing an increase in the *C*_t_ value over 5 and a decrease in cfu ~100 is considered effective (Ma et al., 2022). **Figure 6** shows the percentage of bacterial clearance by the four chimeras relative to the untreated control. UGK-17 was the most effective on *C*Las both by DNA and RNA qPCR. From the DNA qPCR, all four chimeras (30P-3, UGK-9, UGK-13, and UGK-17) appeared to be equally effective on *E. amylovora*. However, the RNA qPCR clearly revealed the higher effectiveness of UGK-13 and UGK-17 compared to UGK-9 and 30P-3. We relied more on the qPCR of RNA to differentiate the effectiveness of the peptides to clear bacteria. **Supplementary Tables SIIIA**, **SIIIB** show the qPCR data for both peptide concentrations 20 and 25 μM. In summary, our data indicated that UGK-17 and UGK-13 were effective in clearing *E. amylovora*, whereas UGK-17 was effective in clearing *C*Las.

**Table 4:** Data from the detached leaf assay on infected citrus (grapefruit) and apple (Red Delicious) leaves measuring the effect of a 0.8-1ml peptide solution at 25 μM concentration.

Treatment	DNA	RNA
*C*Las clearance from infected citrus leaves		
Water (control)	23.99 ± 0.020 (9.4 × 10^5^ ± 0.051)	26.04 ± 0.020 (7.4 × 10^5^ ± 0.071)
UGK-17	31.44 ± 0.013 (9.2 × 10^3^ ± 0.210)	33.30 ± 0.026 (7.0 × 10^2^ ± 0.310)
UGK-9	23.07 ± 0.009 (10.3 × 10^5^ ± 019)	24.56 ± 0.019 (8.8 × 10^5^ ± 0.053)
UGK-13	28.05 ± 0.012 (5.5 × 10^4^ ± 0.065)	31.12 ± 0.011 (7.7 × 10^3^ ± 0.065)
30P-3	29.08 ± 0.018 (2.3 × 10^4^ ± 0.122)	31.80 ± 0.028 (6.8 × 10^3^ ± 0.190)
*Erwinia amylovora* clearance from infected apple leaves		
Water (control)	28.73 ± 0.590 (5.6 × 10^4^ ± 0.07)	28.91 ± 0.990 (5.4 × 10^4^ ± 0.051)
UGK-17	34.46 ± 0.028 (400 ± 0.31)	34.10 ± 0.560 (700 ± 0.210)
UGK-9	35.66 ± 1.43 (~10^2^)	32.62 ± 0.570 (4.7 × 10^3^ ± 0.019)
UGK-13	35.78 ± 2.98 (~10^2^)	34.44 ± 0.620 (300 ± 0.065)
30P-3	36.50 ± 0.89 (~10^2^)	31.54 ± 0.018 (7.8 × 10^3^ ± 0.122)

The levels of bacteria in the DNA/RNA extracted from the infected leaves were measured using qPCR, which provided the C_t_ values for the amplicon generated by the CLas- and E. amylovora-specific primers. The colony-forming units (cfu) of the bacterial load were computed using the cfu vs. C_t_ standard curve (Bao et al., 2020; Salm and Geider, 2004).

CLas, Candidatus Liberibacter asiaticus.

### Host-derived chimeric peptides augment plant innate immunity

A plant’s innate immunity during bacterial infection mainly involves the induction and coordination of pathogen-associated molecular pattern (PAMP)-triggered immunity (PTI) and salicylic acid/jasmonic acid/ethanol (SA/JA/ET) signaling (Nishad et al., 2020). PTI is induced by bacterial PAMP, leading to the activation of transcription factors *via* the mitogen-activated protein kinase (MAPK)/MAPK kinase (MAPKK)/MAPK kinase kinase (MAPKKK) signalosome cascade and, finally, the induction of the PR proteins. However, PTI may be inhibited by the bacterial effectors, which, however, may be countered by effector-triggered immunity (ETI) through the binding of the bacterial effectors to the intracellular Nod-like receptor (NLR) resistance protein (Yuan et al., 2021). Effector–NLR binding may rescue PTI signaling by reinforcing the MAPK/MAPKK/MAPKKK signalosome complex, leading to disease resistance. The universal WRKY transcription factors play a key role in both activating or suppressing specific defense genes (Bakshi and Oelmüller, 2014). SA/JA/ET signaling is an important component of a plant’s innate immunity against bacterial infections (Zhang et al., 2018; Li et al., 2019). The general scheme of this phytohormone signaling involves the activation of inducible transcription factors and the production of PR proteins. Thus, we focused on examining whether the innate immunity in citrus/apple involving PTI, ETI, and SA/JA/ET signaling was augmented during infection by the chimeric peptides. RNA was extracted from both treated and untreated citrus/apple samples from the detached leaf assay described above. Subsequently, differential expression of the selected genes in PTI, ETI, and SA/JA/ET signaling was analyzed using qPCR. It has been proposed that the host antimicrobial peptides may possess the intrinsic ability to directly activate MAPK/MAPKK/MAPKKK and, therefore, the production of the PR proteins (Jain and Khurana, 2018). We did not test this hypothesis in this study by measuring the gene expression in healthy (bacteria-free) citrus or apple leaves upon treatment of the chimeric peptides.

**Figure 7:**
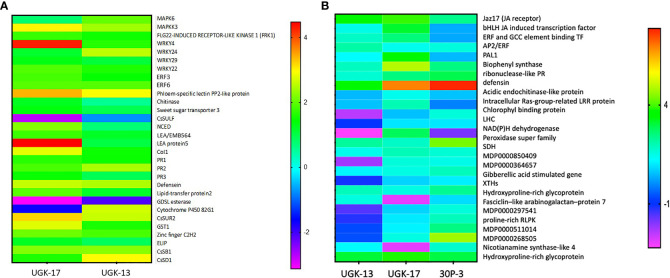
**(A)** Selected genes encoding lipid transfer protein 2, LTP2 (XM_006482145.3); ethylene-responsive transcription factor 3, ERF003 (XM_006483296.3); chitinase (XM_015532796.2); zinc finger; C2H2 type (XM_015531045.2); GDSL esterase (XM_006478917.3); abscisic acid-induced regulated protein (XM_025101123.1); LEA protein 5, LEA5 (NM_001289140.1); cytochrome P450 82G1 (XM_006479159.3); sodium/hydrogen exchanger 2 (XM_006479811.3); phloem-specific lectin PP2-like protein (XM_025095878.1); ethylene-responsive transcription factor 6, ERF006 (XM_006466962.3); sweet sugar transporter 3 (XM_006490501.3); MAPK6 (XM_025097223.1); defensin (XM_006470821.3), EDS (XM_006476627.2); CoI1 (XM_006486308.3); MAPKK3 (XM_006470193.3); WRKY24 (XM_006468068.3), WRKY4 (XM_006483024.2); PR1 (XM_006474081.3); MYB13 (XM_006479482.2); PR2 (KAH9738797.1); and PR3 (KDO71433.1). The selected genes were also shown by RNA sequencing (RNA-seq) to be differentially expressed upon *Candidatus* Liberibacter asiaticus (*C*Las) infection in citrus. The expression of the selected genes in treated and untreated citrus leaves was normalized relative to the expression of the housekeeping gene (*GAPDH*). **(B)** Selected genes encoding Jaz17, jasmonic acid (JA) receptor (MDP0000241358); bHLH, JA-induced transcription factor (MDP0000242554); EBP, ethylene-induced binding GCC element binding transcription factor (MDP0000241358); AP2/ERF, which regulates the biosynthesis of carotenoids by regulating the transcription of *PSY* and *PAL1*; salicylic acid (SA)-inducing PHE ammonia lyase 1 (MDP0000388769); chalcone and stilbene synthase in flavonoid synthesis (MDP0000168735); ribonuclease-like PR (MDP0000782085); apple defensin (MDP0000362305); acidic endochitinase-like protein (MDP0000280265); intracellular Ras group-related LRR protein (MDP0000281307); Chlorophyll binding protein PSII LHC (MDP0000708928); the light-harvesting complex, LHC (MDP0000601491); NAD(P)H dehydrogenase (MDP0000509613); peroxidase superfamily (MDP243237); sorbitol dehydrogenase, SDH-GroES-like zinc-binding alcohol dehydrogenase family protein (MDP0000515106); MDP0000850409; MDP0000364657; gibberellic acid-stimulated *Arabidopsis* (GASA) gene (MDP0000201700); xyloglucan endotransglucosylases/hydrolases, XTH (MDP0000361876); hydroxyproline-rich glycoprotein family protein (MDP0000248516); fasciclin-like arabinogalactan protein 7 (MDC015146.108: 31720–32772); MDP0000297541; proline-rich receptor-like protein kinase (MDP0000511014); MDP0000268505; nicotianamine synthase-like 4 (MDP0000412490); and hydroxyproline-rich glycoprotein family protein: MDP0000248516. The selected genes were also shown by RNA-seq to be differentially expressed upon *Erwinia amylovora* infection in apple. The expression of the selected genes in treated and untreated apple leaves was normalized relative to the expression of the housekeeping *GAPDH* gene.

**Figure 7A** shows the heatmap of the fold changes on a Log_2_ scale of the selected genes from treatment with 25 μM UGK-17 and UGK-13 on infected grapefruit leaves at 3 days posttreatment and absorption of 0.8–1 ml of the peptide solution. The fold changes were normalized relative to the expression of the housekeeping gene, *GAPDH*. UGK-17 at 25 μM cleared *C*Las to almost 100%, whereas UGK-13 at the same concentration cleared over 80%. The selected genes belong to pattern recognition receptors or PRR (FRK1), singling proteins (MAPK6, MAPKK3, CoL1, LEA5, and EMB564), transcription factors (WRKY4/22/24/29, ERF003/6, and zinc fingers), and PR proteins (PR1/2/3, defensin Ec-AMP-D61, chitinase1, and LTP2). The selected list also included detoxifying enzymes such as CYPP450 82G1 and GST1, the glycosidases CsSB1 and CsSD1, and the lipase GDSL, which may be expressed as a defense response. The phloem-specific PP2 protein, a marker for *C*Las infection, was overexpressed. Most of the selected genes were overexpressed (with two-three fold changes) by peptide treatment, most notably the PR proteins, which are the end products of PTI, ETI, and SA/JA/ET signaling.

**Figure 7B** shows the heatmap of the fold changes on a Log_2_ scale of the selected genes from treatment with 25 μM UGK-17, UGK-13, and 30P-3 on infected Red Delicious apple leaves at 48 h posttreatment and absorption of 0.8–1 ml of the peptide solution. The selected genes contained plasma membrane and intracellular receptors, genes such as *LHC* and *Jaz17*; gibberellin (GA)-stimulated genes involved in cytosolic signaling; transcription factors including JA-induced *bHLH*, *ERF*, and *AP2*/*ERF*; and, most importantly, the PR genes [ribonuclease, defensin, chitinase, and the detoxifying enzymes peroxidase and succinate dehydrogenase (SDH)]. In addition, the genes for XTH (xyloglucan endotransglucosylase/hydrolase) and the hydroxyproline-rich protein involved in membrane structure and biogenesis were included. The selected genes have been shown to be important components of the apple reactome (Kamber et al., 2016). Treatment with the three chimeras showed distinctly different patterns in terms of gene expression. A lot more genes were overexpressed by UGK-17 than by the UGK-13 chimera, although both showed similar bactericidal activities (see **Figures 5C, D**). Moreover, 30P-3, which showed lower activity by RNA qPCR, also showed lower expression of the selected genes.

## Discussion

In this paper, we reported the design of the α/β peptides, which are chimeras formed by two different segments. We have previously reported on the design of protein chimeras in which two 5- to 25-kDa host protein domains (instead of the <5-kDa peptides described here) were joined by a linker (Dandekar et al., 2012; Joo et al., 2016). The two host protein domains represented lysis and recognition of the infecting bacteria. Transgenic grape expressing these chimeras showed resistance against PD in the greenhouse and field studies. The combination of molecular modeling and bactericidal/toxicity analyses showed that the chimeric peptides UGK-13 and UGK-17 were the two promising non-toxic candidates in that both of them cleared *E. amylovora* from apple leaves infected with fire blight, whereas UGK-17 cleared *C*Las from citrus infected with HLB. The qPCR also showed that both UGK-13 and UGK-17 augmented the innate immunity in citrus and apple during infection. The bactericidal peptide segments (designated here as single units) in the host proteins were discovered almost three decades ago (Gupta and LLC., 2021). They have been shown to be active against antibiotic-resistant planktonic and biofilm bacteria. In addition, they were expected to exert immune stimulatory activity (Moghaddam et al., 2015). However, it was soon discovered that the bacteria quickly evolved resistance against the host peptide by modifying their membrane structure (Maria-Neto et al., 2015; Joo et al., 2016). As described herein and in [14], our approach of combining two antibacterial peptide segments helped overcome bacterial resistance while retaining bactericidal effects. Finally, the designed chimeras are not toxic to humans and plants and are stimulatory to the host’s innate immune system.

**Figure 8:**
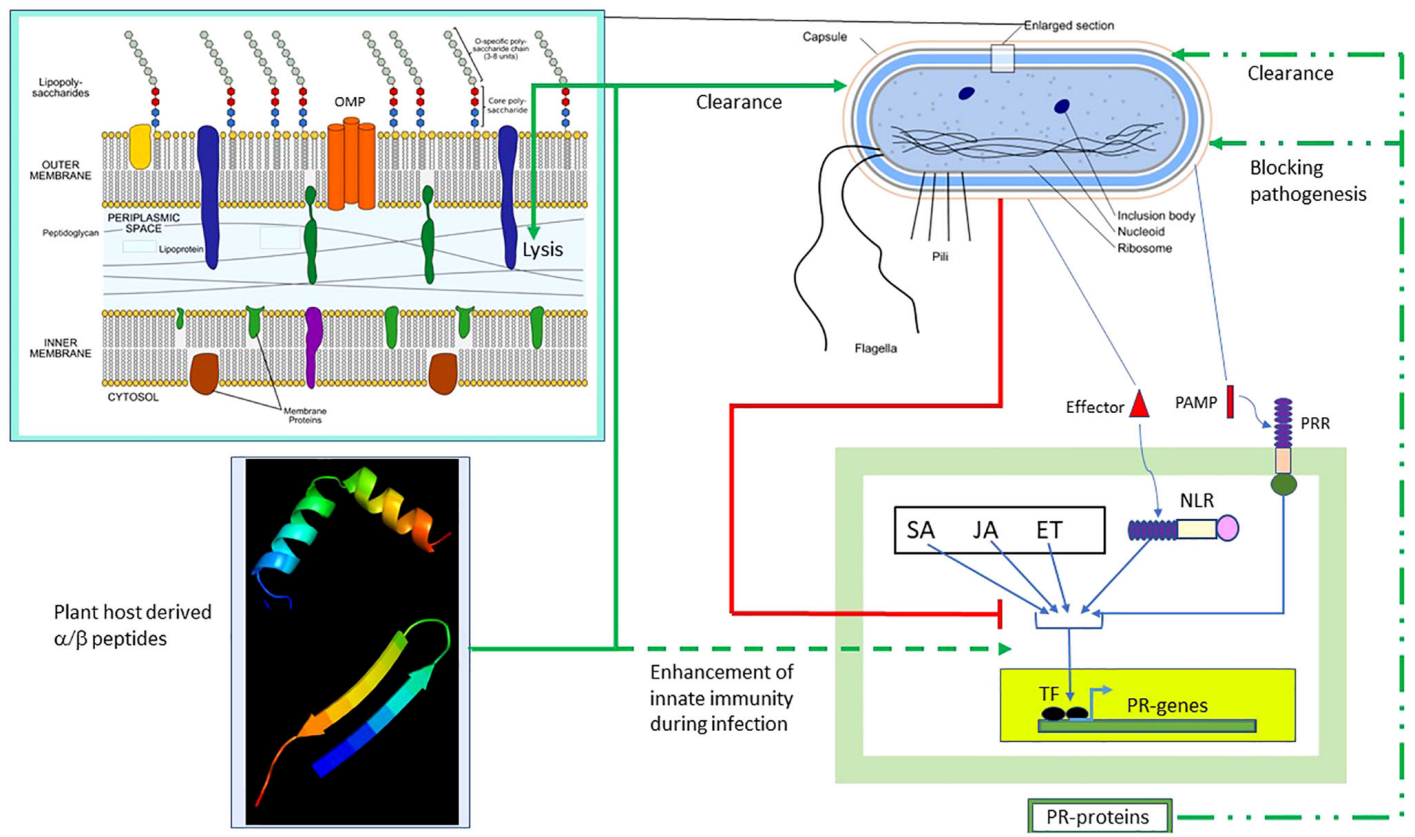
Effect of α/β peptides on plant–bacteria interaction. The schematic representation of the Gram-negative bacterium was adapted from https://en.wikipedia.org/wiki/Gram-negative_bacteria. Several intracellular and membrane-bound bacterial components are shown. The (OM–periplasm–IM) section was enlarged. Bacterial PAMP induces PTI, whereas the effector activates ETI. The SA/JA/ET pathways act in concert with the PT/ETI pathways to induce the TF, leading to the production of pathogenesis-related (PR) proteins capable of clearing bacteria and/or blocking pathogenesis (*broken green lines*). Bacteria employ multiple strategies to block the PTI/ETI/SA/JA/ET pathways (*solid red lines*). The α/β peptides overcome bacterial resistance. They clear bacteria by membrane lysis (*solid green lines*) and also augment the PTI/ETI/SA/JA/ET response during infection (*dashed green lines*). *TF*, transcription factor; *ET*, ethanol; *ETI*, effector-triggered immunity; *IM*, inner membrane; *JA*, jasmonic acid; *NLR*, Nod-like receptor; *PAMP*, pathogen-associated membrane pattern; *PRR*, plasma membrane recognition receptor; *PTI*, PAMP-triggered immunity; *SA*, salicylic acid.

**Figure 8** summarizes the results of our study and the possible scope of α/β chimeric peptides in the treatment of bacterial diseases in plants. The innate immune response is induced in plants during bacterial infection, which, as described above, involves the PTI/ETI and plant hormone SA/JA/ET pathways, leading to the production of PR proteins. The end result is bacterial clearance and/or blocking of bacterial pathogenesis (shown respectively as broken green lines). However, pathogenic bacteria have evolved multiple strategies (Jones and Dangl, 2006) to block the PTI/ETI and plant hormone SA/JA/ET pathways, as well as the resulting production of PR proteins (shown as red lines). We presented a counterstrategy to overcome bacterial resistance by designing host-derived and non-toxic α/β chimeric peptides that lyse the membrane and clear the pathogenic bacteria (shown as solid green lines), as well as augment the PTI/ETI and plant hormone SA/JA/ET pathways (shown as dashed green lines), during bacterial infection. Our strategy can be extended to the design of α/β chimeric peptides against other bacterial diseases in plants with agricultural and economic importance.

## Experimental procedures

### Phytotoxicity assays in plants

The leaves of different plants were infiltrated with 10 μl of each peptide at different concentrations using a syringe. PBS was used as the negative control. Two independent experiments were performed in which three leaves were inoculated abaxially/adaxially at three different points. The necrotic effects were visually monitored to examine the possible toxicity of the peptide. The leaves were placed in agar plates (1%) for 7 days and maintained under controlled conditions at 26°C and a photoperiod of 16-h light/8-h dark.

### Minimal inhibition concentration assay

Bacterial cultures of *E. coli* BL21 and ATCC 25922 were inoculated into 20 ml Mueller Hinton II broth (MHB, Difco, Franklin Lakes, NJ, USA) and incubated for 16 h at 37°C with shaking. The concentration of the bacterial cells was determined using an OD_600_ = 1 for 10^9^ cells and was verified by plating corresponding dilutions. Diluted aliquots (10^5^ cells/ml) were sub-cultured in 96-well polystyrene microtiter plates with twofold serial dilutions of the corresponding peptides (20–0.16 µM) and then incubated for 16 h at 37°C without shaking. After the incubation, the ODs of the plates were measured using a plate reader (Synergy HTX; BioTek, Santa Clara, CA, USA). The MIC was defined as the lowest concentration of peptides that produced no visible growth (Wiegand et al., 2008).

### Bioluminescence assay

The BacTiter-Glo™ Microbial Cell Viability Assay (Promega G8231, Madison, WI, USA) was used to determine the number of viable microbial cells (or cfu) in the cultures obtained for the determination of MICs. After incubation with peptides and determining the ODs, the bacterial cultures in polystyrene plates were carefully resuspended to homogeneity by shaking in a plate reader incubator for 5 min at 100 rpm. Of the corresponding bacterial cultures, 50 µl was transferred into a black microtiter plate and was mixed with an equal volume of the BacTiter-Glo™ reagent, which resulted in bacterial lysis and generated a bioluminescence signal proportional to the ATP content. To determine the exact cfu values, a standard curve was used to correlate the cfu to bioluminescence. Bioluminescence was measured using a plate reader (Synergy HTX; BioTek). The dose–response curves were obtained for the most active peptides by plotting the cfu values against the peptide concentrations. To determine the inhibitory concentration, IC_50%_ and IC_99%_, values, the dose–response curves were fitted to Hill’s equation (Gadagkar and Call, 2015).

### Staining of live/dead cells

To examine the peptide-induced bacterial death, the LIVE/DEAD cell staining kit (L7012; Thermo Fisher, Waltham, MA, USA) was used. An overnight bacterial culture of *E. coli* BL21 was diluted in fresh 1:10 Luria–Bertani (LB) medium and bacterial growth continued for 2 h at 37°C with aeration at 200 rpm. Of the bacterial culture, 10 ml was precipitated by centrifugation at 5,000 rpm for 15 min. The bacteria were resuspended in 2 ml of 0.15 M NaCl. Three additional washes with 0.15 M NaCl were performed to remove traces of bacterial media. The bacterial concentration was adjusted with PBS to obtain a final concentration of 5 × 10^6^ cells/ml. The bacterial suspension was then mixed with the peptide solution in 0.15 M NaCl and incubated for 1 h at room temperature. Thereafter, the cells were stained with a LIVE/DEAD dye mix. The fluorescence of live cells showed green due to the SYTO™ 9 nucleic acid. The SYTO 9 stain generally labels all bacteria in a population: those with intact membranes and those with damaged membranes. The fluorescence of dead cells showed red since PI penetrates only those bacteria with damaged membranes, causing a reduction in the SYTO 9 fluorescence when both dyes are present. For the live cell control, the cells were incubated with PBS only; for the dead cell control, the cells were killed with 70% isopropanol before staining.

The live/dead cell ratio was measured according to the manufacturer (L7012; Thermo Fisher). The cells were treated with peptides, as described above. At the end of the incubation, the peptide-treated bacterial suspensions were mixed with equal volumes of 2× working solution of the LIVE/DEAD dyes. The samples were then incubated for 15 min at room temperature in the dark. The fluorescence intensity was measured using a plate reader (SpecraMax M4; Molecular Devices, Sunnyvale, CA, USA) in 96-well black microtiter plates (Corning, Corning, NY, USA): emission 1, green: *Λ*_ex_ = 485 nm, *Λ*_em_ = 530 nm; emission 2, red: *Λ*_ex_ = 485 nm, *Λ*_em_ = 630 nm. The ratio was determined and normalized using the control samples.

### Hemolytic assay

To determine the toxicity of the peptides, a hemolytic assay was routinely performed using human erythrocytes obtained as 10% Single Donor Human Red Blood Cells Washed (Innovative Research, Clearwater, FL, USA). The procedure was based on the measurement of hemoglobin release upon erythrocyte lysis. PBS (pH 7.4) was used to suspend the erythrocytes and dilute the peptide samples. Human erythrocytes (red blood cells, RBCs) were washed with PBS and adjusted to a concentration of 1% (*v*/*v*). Approximately 100 µl of 1% RBC was then mixed with 100 µl of the peptide samples and the tubes were incubated at 37°C for 60 min. The samples were centrifuged for 5 min at 14,000 × *g*, the supernatants were collected, and the ODs at 445 and 415 nm corresponding to the Soret bands of the released hemoglobin were determined by NanoDrop. PBS and 0.01% Triton-X-100 were used respectively as the negative (0%) and positive (100%) controls (Sineva et al., 2009).

### MTT cytotoxicity assay

The MTT assay (Denizot and Lang, 1986) was used to determine the cytotoxicity of the compounds by examining the cell metabolic activity, e.g., the activity of NAD(P)H-dependent cellular oxidoreductases is proportional to the number of viable cells present. The cells were seeded at a density of 1 × 10^4^ cells/well in 96-well culture plates and allowed to adhere overnight at 37°C. After 24 h of incubation, HEK 294 cells (Sigma-Aldrich, St. Louis, MO, USA) were treated with 20 µM of peptides and incubated for 72 h. PBS (pH 7.4), the solvent used to make the peptide stock solution, was used as a non-treatment control in this experiment. At 72 h post-stimulation, the cells were treated with 10 μl of 5 mg/ml MTT solution (Sigma-Aldrich) and incubated for an additional 3 h. Triton X-100 (0.1%) was used as 100% control. Formazan crystals were dissolved in 100 μl of lysis solution, and the absorbance was determined at 570 nm using a microplate reader (Synergy HTX; BioTek).

### Monitoring of bacterial lysis

To monitor bacterial lysis during the bacterial peptide treatments, we transformed *E. coli* BL21 with reporter plasmids expressing codon-optimized eGFP and NanoLuc^®^ Luciferase (Promega, Madison, WI, USA) genes under the control of the constitutive P*upp* promoter. To monitor bacterial lysis using fluorescent microscopy, BL21 (pACYC184-GFP) cells were incubated with 10 µM of the antibacterial peptides, and microscopy was performed at time points 15, 30, and 60 min and at 4 h. All fluorescence images were taken at 488 nm excitation. The emission filter was 525/50 nM for cytoplasmic GFP. Phase-contrast images were also collected at the same time points. To monitor bacterial lysis in solutions by protein leakage, BL21 (pACYC184 nLuc) cells were washed with PBS (pH 7.4) three times, diluted with PBS to 10^5^ cfu/ml, and then incubated with peptides for 15, 30, and 60 min. After incubation, the intact bacterial cells were precipitated and the luciferase activity of the supernatant was examined using the Nano-Glo^®^ Luciferase Assay Substrate (Promega). The linearity of the assay was assessed using activity measurements of serial dilutions of the completely lysed BL21 (pACYC184 nLuc) bacterial culture. An undiluted bacterial culture was taken as 100%. Data were corrected to the background luciferase activity obtained with the supernatant of untreated cells.

### Detached leaf assay

Both uninfected and infected citrus (grapefruit) leaves were obtained from Texas A&M University—Kingsville Citrus Center, Weslaco, TX, while the infected and uninfected apple (Red Delicious) leaves were obtained from Las Cruces, NM (i.e., Burke Apple Orchard). The leaf samples were stored at −80°C in sealed in Ziplock bags. Before the experiment, the Ziplock bags were placed in a box and transferred into a −20°C refrigerator. Before treatment, the leaves were thawed and dipped in 1–2 ml peptide solution at the specified concentration at room temperature for 48–96 h in a biosafety cabinet. The leaves remained dipped until 0.8–1 ml of the peptide solution was absorbed. The leaves were then crushed in liquid nitrogen inside the biosafety cabinet using a mortar and pestle. The crushed leaves were split into two halves: one for DNA and the other for RNA extraction as per the instructions in the E.Z.N.A.^®^ Plant DNA DS Kit (RNeasy plant mini kit). The extracted DNA and RNA were analyzed using qPCR in a BSL-1 laboratory. The forward and reverse primers for the detection of *C*Las were GTCGAGCGCGTATGCAATACG and CTACCTTTTTCTACGGGATAACGC, which were chosen to amplify the 16S DNA/RNA (Bao et al., 2020). The forward and reverse primers for the detection of *E. amylovora* were CACTGATGGTGCCGTTG and CGCCAGGATAGTCGCATA, which were chosen to amplify the locus in the pEA29 plasmid (Santander et al., 2019).

## Data availability statement

The original contributions presented in the study are included in the article/**Supplementary Material**, further inquiries can be directed to the corresponding author.

## Author contributions

SB, ES, LN, NS, and MS performed the experiments, analyzed the data, and drafted the manuscript. GG supervised the experiments, drafted the manuscript, and arranged the funding. MK and JP shipped the samples for the experiments to be conducted in New Mexico and assisted in correcting the manuscript. All authors contributed to the article and approved the submitted version.
