# Supplementary figures and images for: Host-derived chimeric peptides clear the causative bacteria and augment host innate immunity during infection: A case study of HLB in citrus and fire blight in apple
Source: Front Plant Sci. 2022 Dec 23;13:929478. doi: 10.3389/fpls.2022.929478 (PMC9816411; doi:10.3389/fpls.2022.929478)

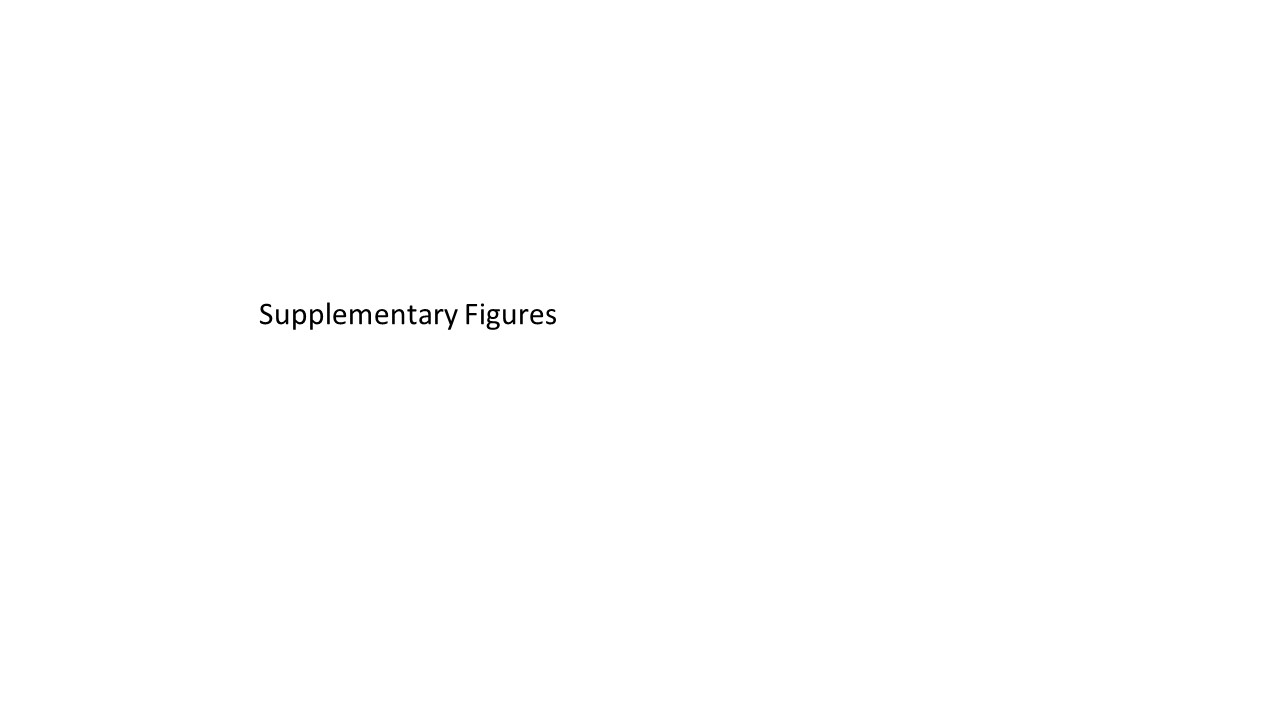

Supplement: Supplementary file 1 [file Image_1.jpeg]

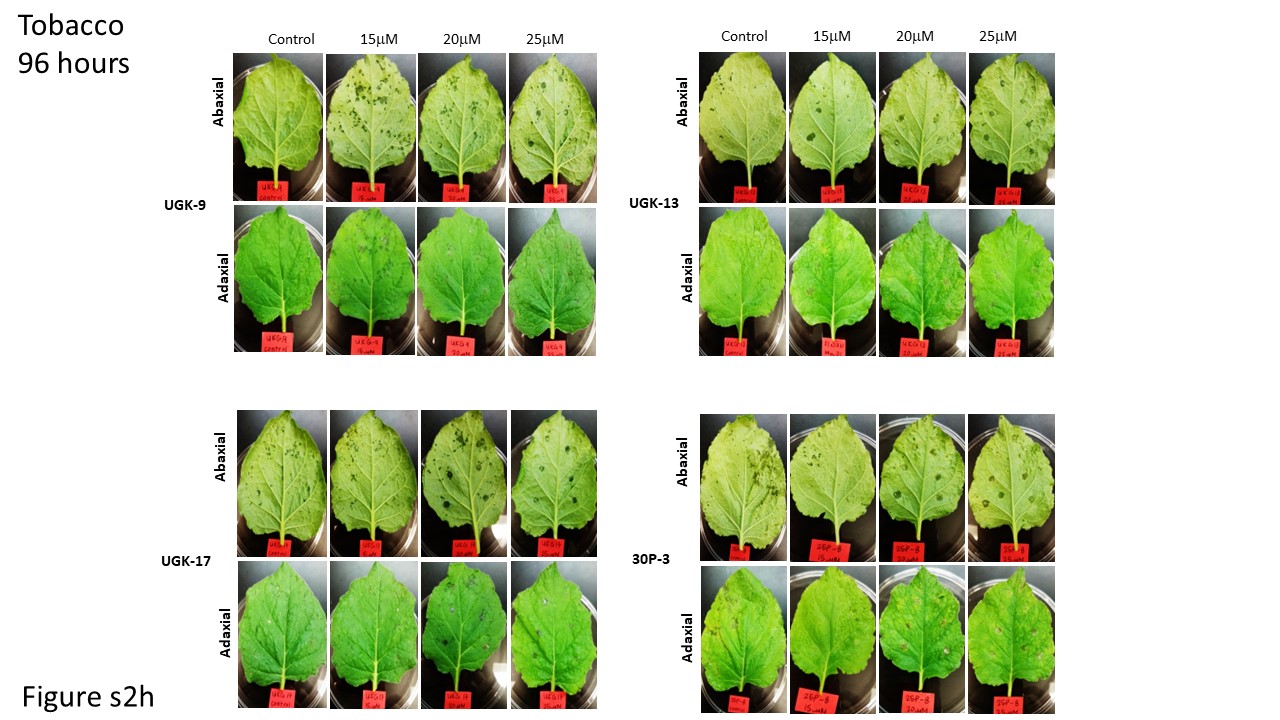

Supplement: Supplementary file 2 [file Image_2.jpeg]

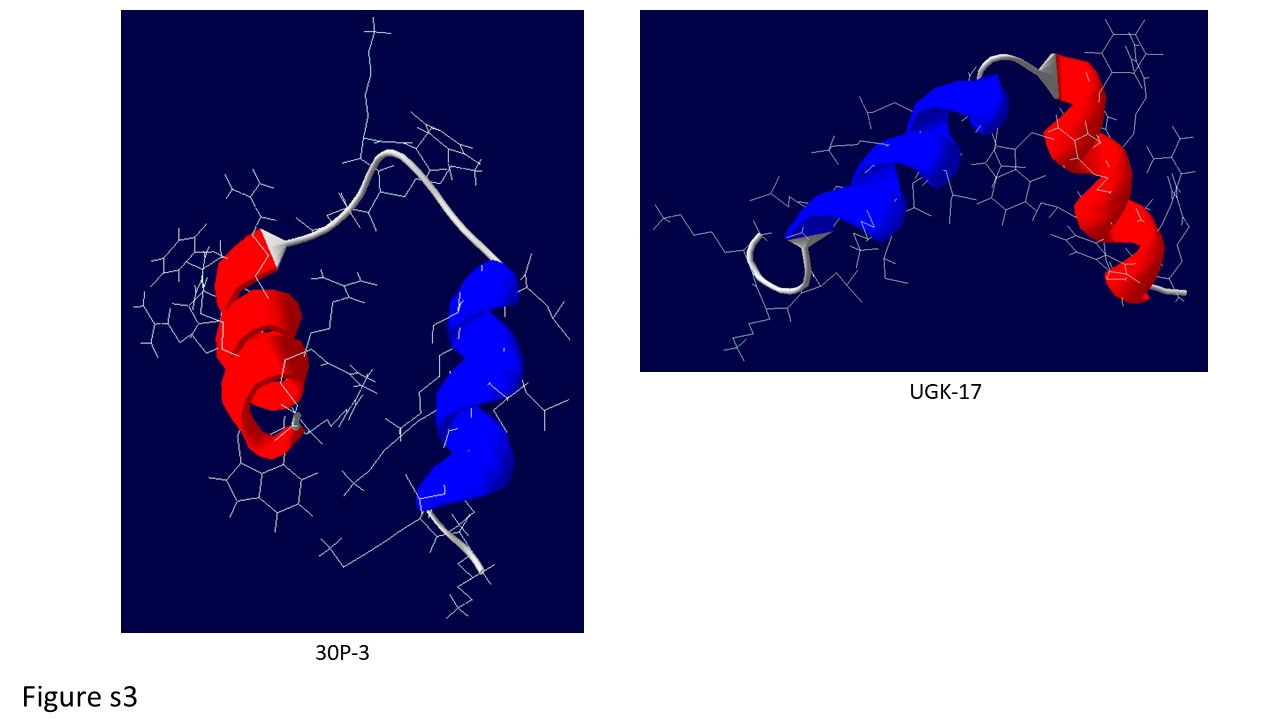

Supplement: Supplementary file 3 [file Image_3.jpeg]

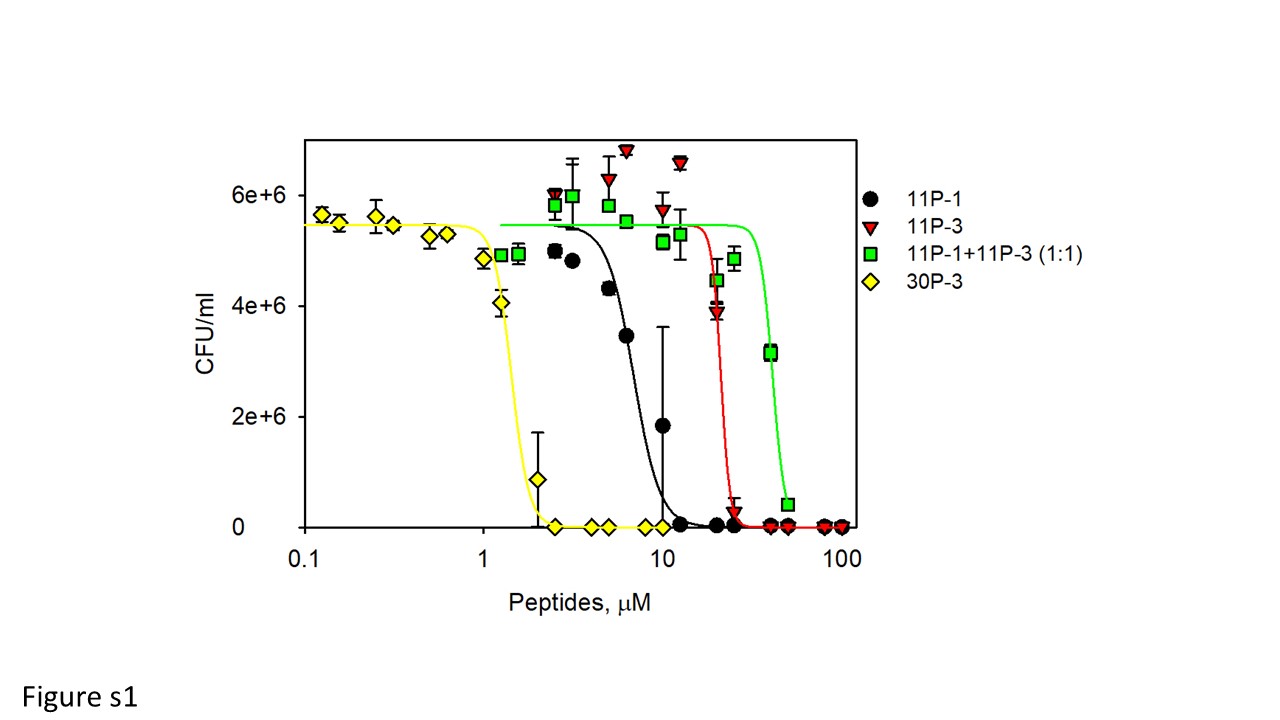

Supplement: Supplementary file 4 [file Image_4.jpeg]

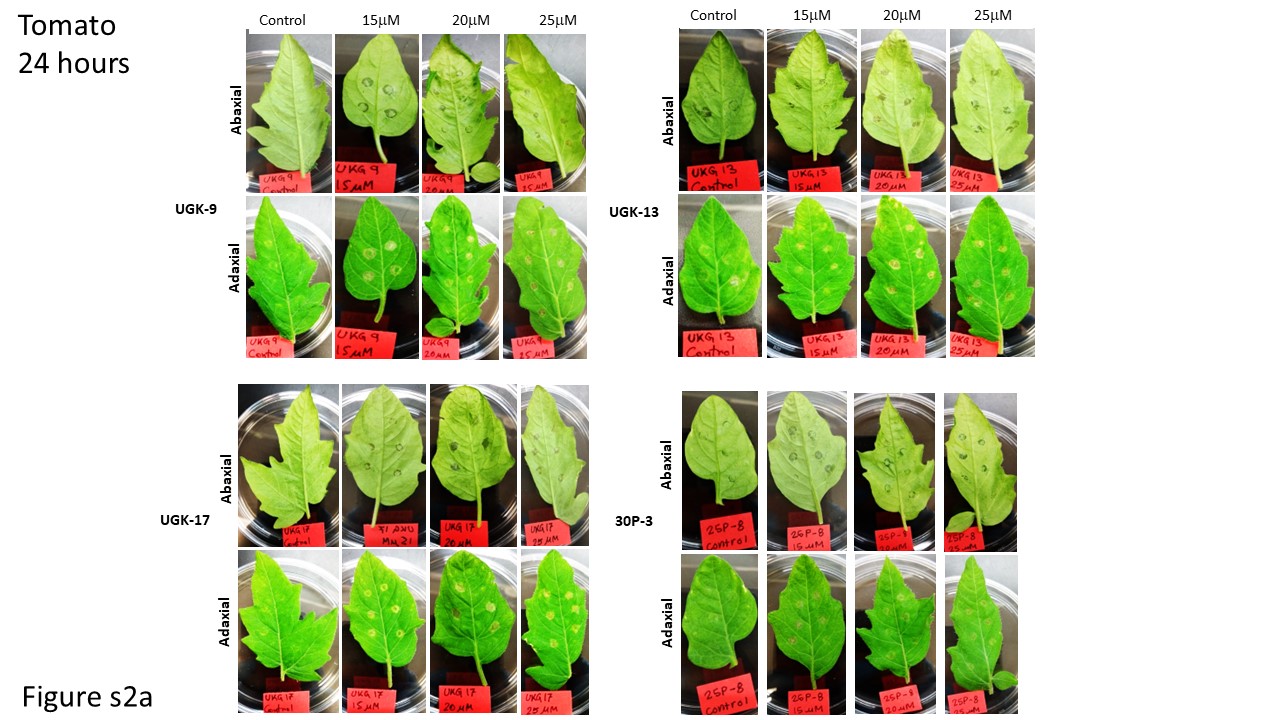

Supplement: Supplementary file 5 [file Image_5.jpeg]

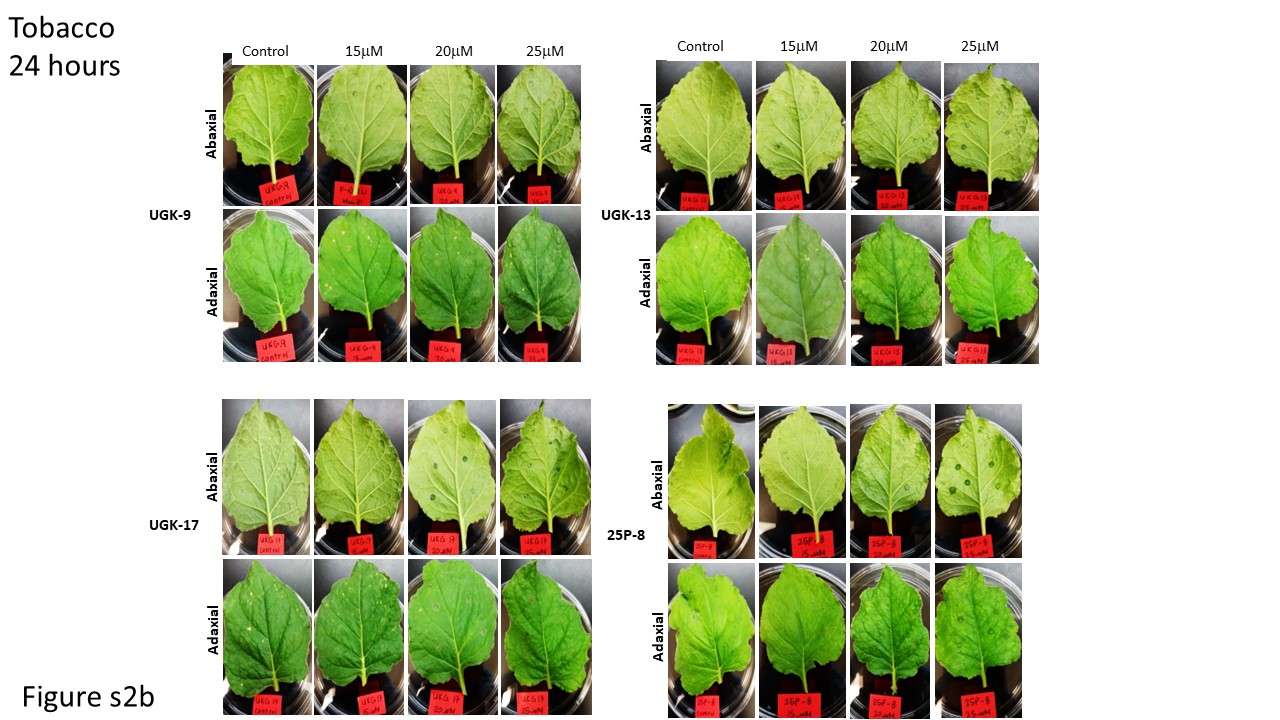

Supplement: Supplementary file 6 [file Image_6.jpeg]

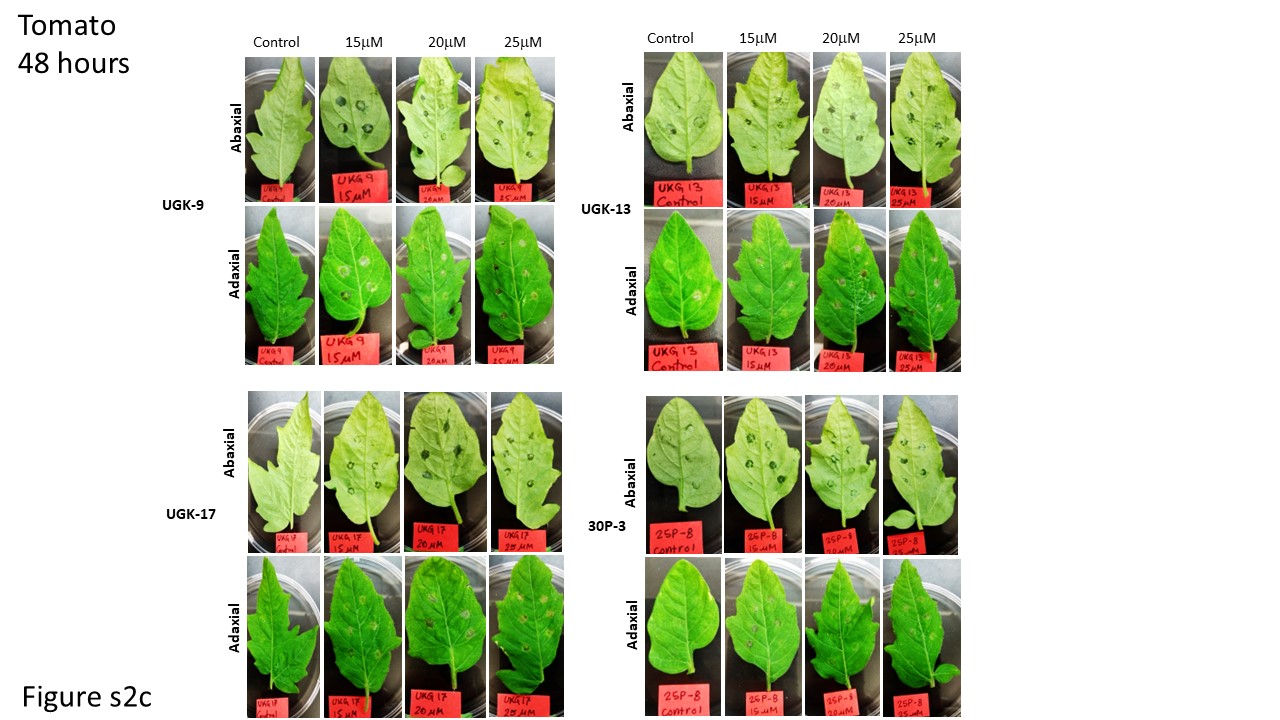

Supplement: Supplementary file 7 [file Image_7.jpeg]

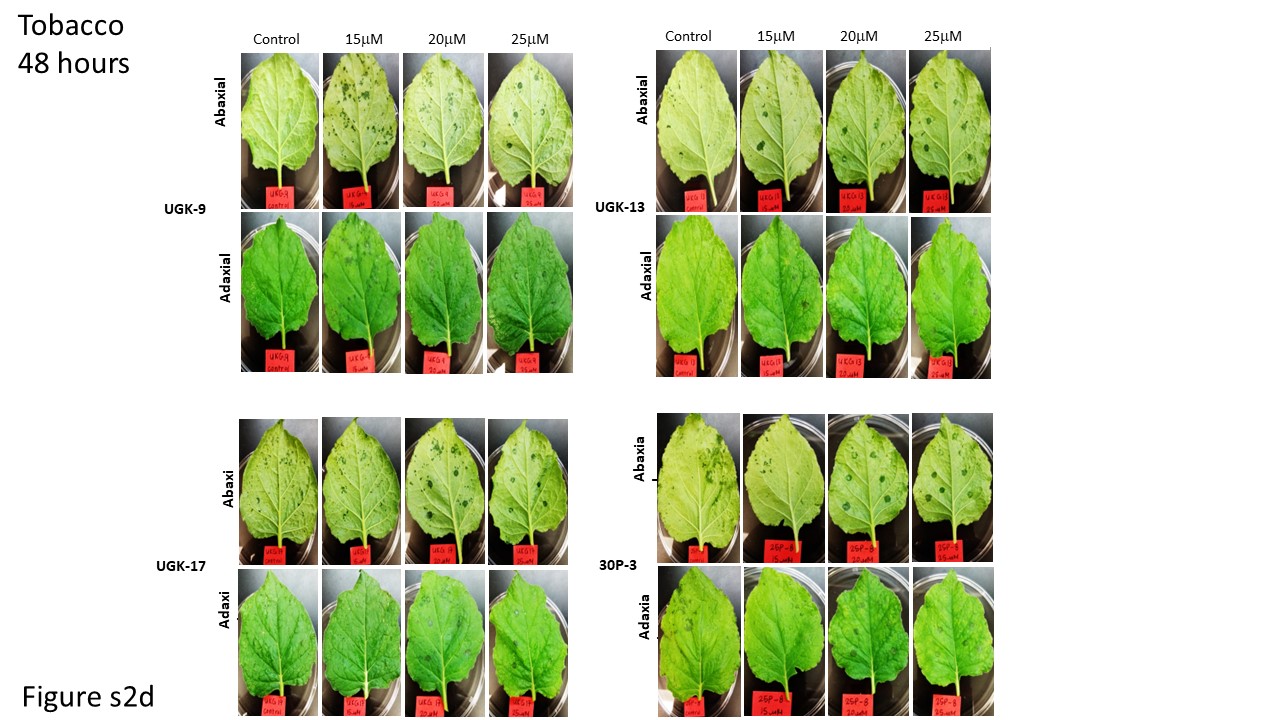

Supplement: Supplementary file 8 [file Image_8.jpeg]

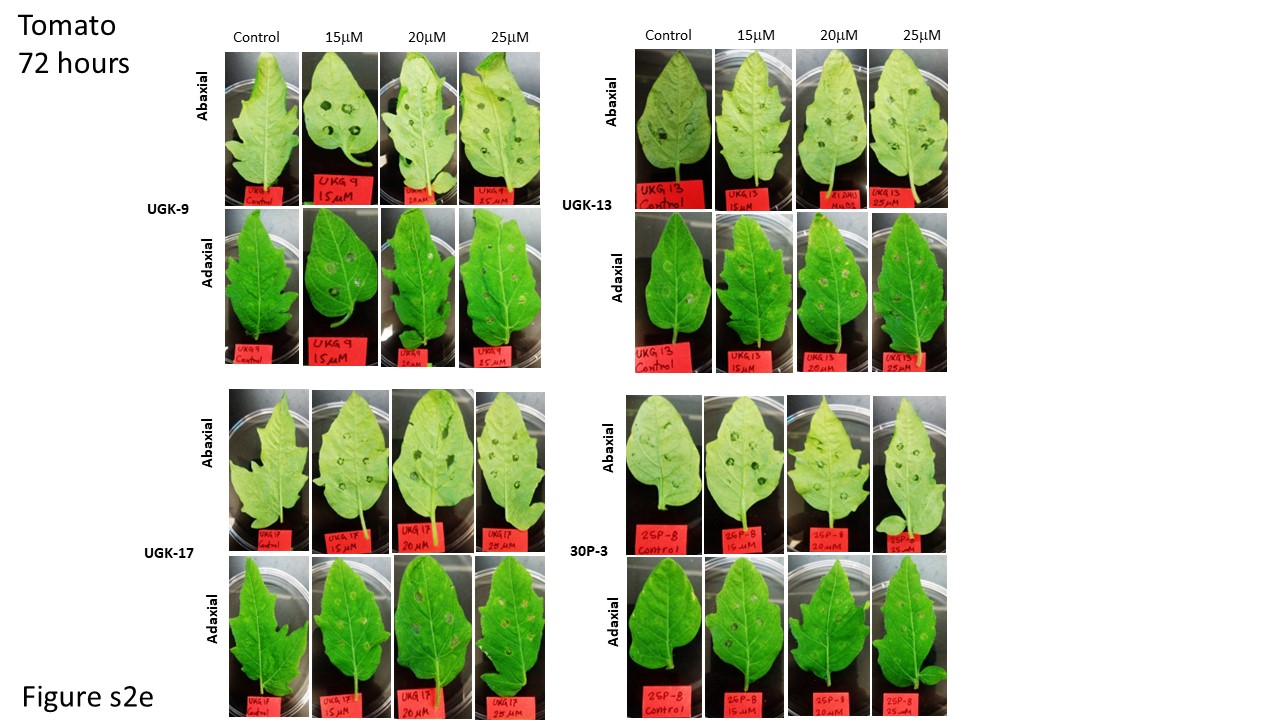

Supplement: Supplementary file 9 [file Image_9.jpeg]

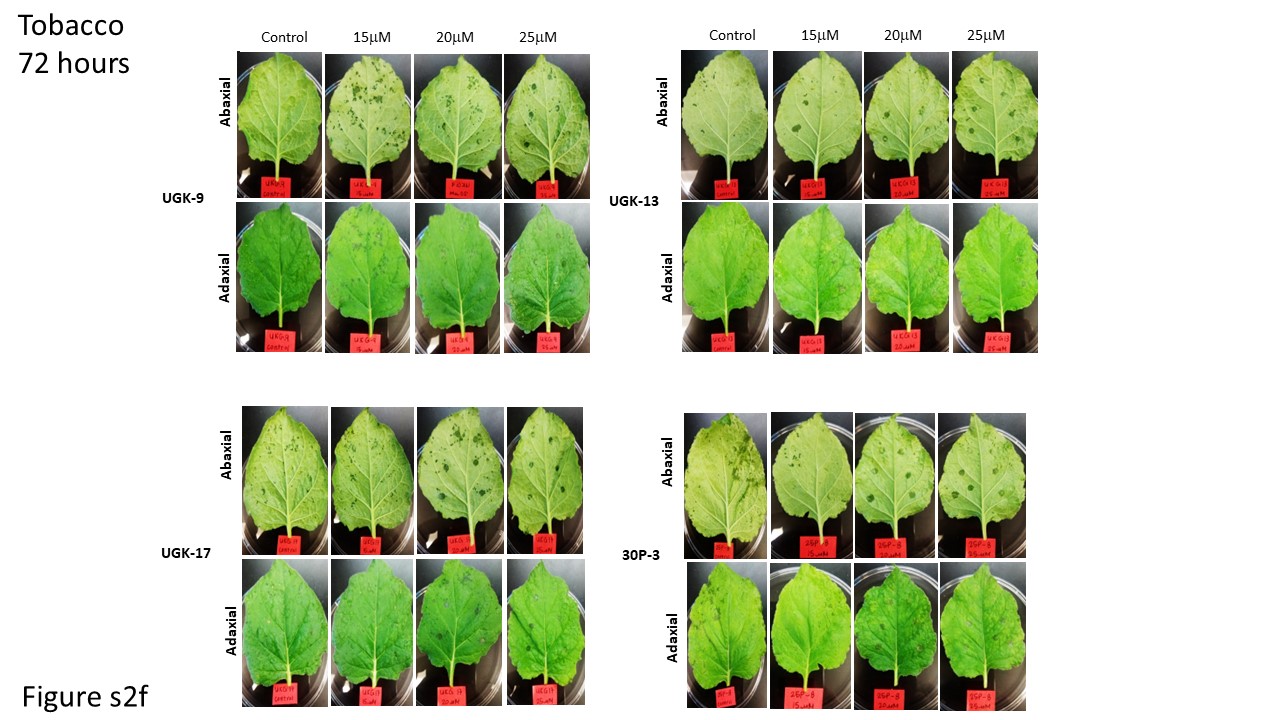

Supplement: Supplementary file 10 [file Image_10.jpeg]

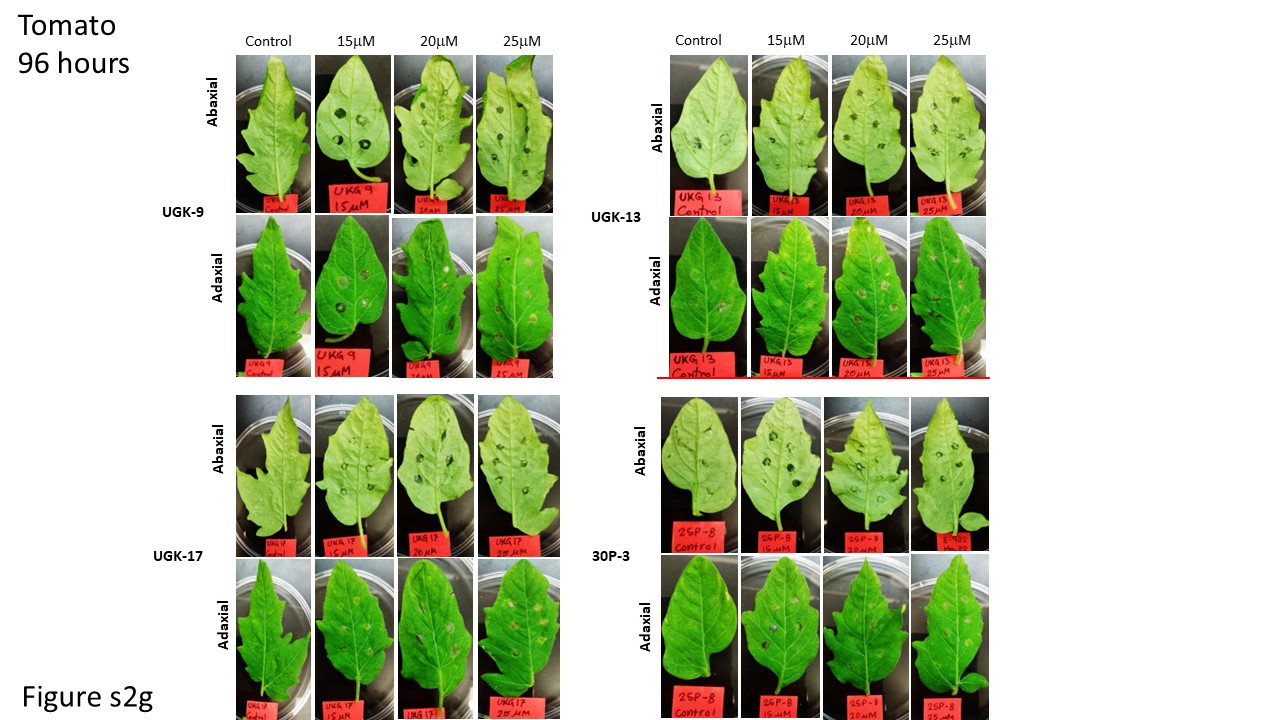

Supplement: Supplementary file 11 [file Image_11.jpeg]
