# Supplementary material for: Host-derived chimeric peptides clear the causative bacteria and augment host innate immunity during infection: A case study of HLB in citrus and fire blight in apple
Source: Front Plant Sci. 2022 Dec 23;13:929478. doi: 10.3389/fpls.2022.929478 (PMC9816411; doi:10.3389/fpls.2022.929478)
Supplement: Supplementary file 12 [file Table_1.docx]

**Legends to supplementary Figures and Tables.**

**Figure s1.** Bactericidal activity of 11P-1, equimolar 11P-3, 11P-1:11P-3, and the chimera 30P-3 on *E. coli* BL21 as determined by the bioluminescence assay. Note that the MIC of the equimolar mixture of 11P-1 and 11P-3 is higher than that of the individual units. Mutual repulsion 11P-1 (with +5 charge) and 11P-3 (with +6 charge) may hinder their simultaneous access to the bacterial membrane. However, in the chimeras such as 30P-3 and UGK-17 most of the positive charges are disposed away from each other or screened by negatively charged or hydrophobic residues. See Figure s3 below.

**Figures s2a-h.** Toxicity analysis by infiltration of tomato and tobacco leaves at 15-25mM peptide concentration. The corrosive effect on leaves was monitored for 96 hours.

**Figure s3.** Spatial disposition of basic (cyan), acidic (purple), and hydrophobic (gray) amino acids (shown as ball and stick) in the energy minimized models of 30P-3 and UGK-17. Most of the positive charges are disposed away from each other in 30P-3 whereas some are screened by hydrophobic residues in between the two basic residues. Most of the positive charges are further disposed away from each other in UGK-17 than in 30P-3; also, the acidic amino acids stabilize the N-terminal (blue) helix through favorable electrostatic interactions and the hydrophobic residues in the interior stabilize the chimeric scaffold.

**Tables sIa and sIb.** Homology of the chimeras with (a) citrus protein and (b) apple proteins.

**Tables sIIa and sIIb.** Ct values for DNA and RNA qPCR for bacteria-specific locus and host-specific reference genes.

**Tables sIa**

**Citrus homologs**

| 1. Alignment statistics for the chimeras AC |
| --- |
| 1. N-terminal fragment   AMP N domain-containing protein [Citrus sinensis]; GenBank: KAH9762819.1  Score Expect Identities Positives Gaps  31.6 bits(67) 0.26 11/14(79%) 11/14(78%) 2/14(14%)  Query 2 KLPEKILKILESLK 15  KL EKI ILESLK  Sbjct 332 KLAEKI--ILESLK 343 Middle partputative disease resistance protein RGA3 [Citrus clementina]; NCBI Reference Sequence: XP_024036637.1Score Expect Identities Positives Gaps26.5 bits(55) 14 8/8(100%) 8/8(100%) 0/8(0%)Query 10 ILESLKGS 17ILESLKGSSbjct 267 ILESLKGS 274  1. 25/30 amino acid length of the chimeras AC  Extra-large guanine nucleotide-binding protein 3 [Citrus sinensis]; GenBank: KAH9687262.1 Score Expect Identities Positives Gaps  30.3 bits(64) 0.71 13/25(52%) 16/25(64%) 7/25(28%)  Query 1 KKLPEKILKILESLKGSPGFWQRRI 25  KKL EK +K L+SLK QRR+  Sbjct 503 KKL-EKLMKQLQSLK------QRRV 520   1. C-terminal part  protein kinase domain-containing protein [Citrus sinensis]; GenBank: KAH9801395.1 Score Expect Identities Positives Gaps  25.2 bits(52) 38 7/8(88%) 7/8(87%) 1/8(12%)  Query 20 FWQRRIRR 27  FWQ RIRR  Sbjct 695 FWQ-RIRR 701 |
| 1. B. Alignment statistics for the chimeras CA |
| 1. 1. N-terminal plus linker  Valine--tRNA ligase 1 [Citrus sinensis]; GenBank: KAH9646357.1 Score Expect Identities Positives Gaps  26.1 bits(54) 20 8/10(80%) 8/10(80%) 0/10(0%)  Query 6 IRRWRRGSGY 15  I RWRR SGY  Sbjct 290 IIRWRRMSGY 299 hypothetical protein CISIN_1g0401331mg, partial [Citrus sinensis]; GenBank: KDO48896.1 Score Expect Identities Positives Gaps  26.9 bits(56) 10 10/17(59%) 10/17(58%) 4/17(23%)  Query 1 FW---QRRIRRWRRGSG 14  FW Q R RR R GSG  Sbjct 816 FWRIRQKR-RRRRKGSG 831   1. 2. C-terminal part   peptidyl-prolyl cis-trans isomerase FKBP62 [Citrus sinensis]; GenBank: KAH9770943.1  Score Expect Identities Positives Gaps  26.9 bits(56) 10 8/9(89%) 8/9(88%) 0/9(0%)  Query 20 KKLIKKILK 28  KK IKKILK  Sbjct 270 KKVIKKILK 278   1. 3. Linker plus the C-terminal part  hypothetical protein CUMW_263420 [Citrus unshiu]; GenBank: GAY68349.1 Score Expect Identities Positives Gaps  25.7 bits(53) 26 8/11(73%) 9/11(81%) 0/11(0%)  Query 17 SPGKKLIKKIL 27  SP KKL+KK L  Sbjct 11 SPNKKLVKKFL 21   1. 4. Linker  hypothetical protein CISIN_1g0415571mg, partial [Citrus sinensis]; GenBank: KDO48753.1 Score Expect Identities Positives Gaps  26.9 bits(56) 9.1 8/9(89%) 8/9(88%) 0/9(0%)  Query 10 RRGSGYGSP 18  RRGS YGSP  Sbjct 32 RRGSSYGSP 40 |
| 1. . Alignment statistics for the chimeras CD |
| 1. C-terminal   protein DETOXIFICATION 19 isoform X3 [Citrus clementina]; Sequence ID: XP_024033441.1  Score Expect Identities Positives Gaps  26.5 bits(55)14 6/7(86%) 6/7(85%) 0/7(0%)  Query 23 RRWWRWW 29  RRWWR W  Sbjct 31 RRWWRNW 37   1. Linker plus C-terminal   histone deacetylase 15 isoform X1 [Citrus sinensis]  Sequence ID: XP_006485755.1  Score Expect Identities Positives Gaps  32.9 bits(70)0.095 9/17(53%) 10/17(58%) 2/17(11%)  Query 12 IRKVRRPDSPGRRWWRW 28  I K+RR D P WW W  Sbjct 581 IKKIRRADAP--IWWKW 595   1. N-terminal plus linker   Subtilisin-like protease SBT6.1 [Citrus clementina]; Sequence ID: XP_006451169.  Score Expect Identities Positives Gaps  26.9 bits(56) 7.8 10/17(59%) 10/17(58%) 4/17(23%)  Query 1 FW---QRRIRRWRRGSG 14  FW Q R RR R GSG  Sbjct 1008 FWRIRQKR-RRRRKGSG 1023 |
| 1. Alignment statistics for the chimeras DC |
| 1. 1. N terminal  protein DETOXIFICATION 19 isoform X1 [Citrus clementina]; NCBI Reference Sequence: XP_024033440.1 Score Expect Identities Positives Gaps  28.2 bits(59) 2.8 7/8(88%) 7/8(87%) 0/8(0%)  Query 1 RRWWRWWR 8  RRWWR WR  Sbjct 33 RRWWRKWR 40   1. Middle part (with linker)   Flowering locus K domain [Citrus sinensis]  Sequence ID: KAH9699095.1  Score Expect Identities Positives Gaps  26.5 bits(55) 14 8/9(89%) 8/9(88%) 0/9(0%)  Query 9 GSYGSVYGS 17  G YGSVYGS  Sbjct 513 GGYGSVYGS 521   1. Linker plus C-terminal   chaperone protein ClpB1 [Citrus sinensis]  Sequence ID: XP_006477653.  Score Expect Identities Positives Gaps  26.9 bits(56) 10 9/19(47%) 10/19(52%) 5/19(26%)  Query 10 SYGSVYGSPGFWQRRIRRW 28  SY +YG R IRRW  Sbjct 809 SYDPIYGA-----RPIRRW 822 |
| 1. E. Alignment for the S-S bridged L19GA |
| 1. 13 out of 19   Endonuclease [Citrus sinensis]; Sequence ID: KAH9697901.  Score Expect Identities Positives Gaps  23.1 bits(47) 67 9/13(69%) 9/13(69%) 3/13(23%)  Query 4 RLCYKQRCVTYCR 16  RLC RCVT CR  Sbjct 681 RLC--GRCVT-CR 690 |
| 1. N-terminal   Cyclic nucleotide-gated ion channel 1 [Citrus sinensis]; Sequence ID: KAH9646573.1  Score Expect Identities Positives Gaps  27.8 bits(58) 1.5 7/7(100%) 7/7(100%) 0/7(0%)  Query 3 RRLCYKQ 9  RRLCYKQ  Sbjct 517 RRLCYKQ 523 |
| 1. C-terminal   Timeless family protein [Citrus sinensis]; Sequence ID: KAH9677436.1  Score Expect Identities Positives Gaps  23.1 bits(47) 67 6/7(86%) 6/7(85%) 0/7(0%)  Query 9 QRCVTYC 15  QRCV YC  Sbjct 40 QRCVEYC 46 |

**Table sIb**

**Apple homologs**

| 1. Alignment statistics for the chimeras AC |
| --- |
| 1. N-terminal plus linker   hypothetical protein DVH24_018081 [Malus domestica]; GenBank: RXI06039.1  Score Expect Identities Positives Gaps  29.9 bits(63) 0.26 12/17(71%) 13/17(76%)3/17(17%)  Query 2 KLPEKILKILESLK-GS 17  KL EK+ ILESLK GS  Sbjct 304 KLAEKV--ILESLKRGS 318   1. 22 out of 30 residues   hypothetical protein DVH24_018947 [Malus domestica]; GenBank: RXH71592.1  Score Expect Identities Positives Gaps  25.2 bits(52) 9.8 13/25(52%) 14/25(56%) 7/25(28%)  Query 5 EKILK--ILE-SLKGSPGFWQRRIR 26  +K LK ILE L GSPG RR  Sbjct 288 DKELKWHILEIPLNGSPG----RLR 308   1. C-terminal   dnaJ protein ERDJ3B [Malus domestica]; NCBI Reference Sequence: XP_008386734.2  Score Expect Identities Positives Gaps  25.2 bits(52)9.9 7/10(70%) 7/10(70%) 0/10(0%)  Query 21 WQRRIRRWRR 30  W RR RR RR  Sbjct 113 WRRRRRRRRR 122  protein SUPPRESSOR OF npr1-1, CONSTITUTIVE 1-like [Malus domestica]; NCBI Reference  Sequence: XP_008382508.3  Score Expect Identities Positives Gaps  25.7 bits(53)7.1 7/10(70%) 7/10(70%) 0/10(0%)  Query 19 GFWQRRIRRW 28  GFWQR R W  Sbjct 612 GFWQRQQRWW 621 |
| 1. Alignment statistics for the chimeras CA |
| 1. N-terminal plus linker   LOW QUALITY PROTEIN: subtilisin-like protease SBT6.1 [Malus domestica]  Sequence ID: XP_008361242.  Score Expect Identities Positives Gaps  26.9 bits(56)2.7 10/17(59%) 10/17(58%) 4/17(23%)  Query 1 FW---QRRIRRWRRGSG 14  FW Q R RR R GSG  Sbjct 1021 FWRIRQKR-RRRRKGSG 1036   1. Linker plus C-terminal   probable E3 ubiquitin-protein ligase BAH1-like 1 [Malus domestica]  Sequence ID: XP_028954001.  Score Expect Identities Positives Gaps  24.0 bits(49)26 10/14(71%) 10/14(71%) 4/14(28%)  Query 18 PG---KKLIKKILK 28  PG KKL KKILK  Sbjct 19 PGVGFKKL-KKILK 31  TMV resistance protein N-like [Malus domestica]; Sequence ID: XP_028945095.  Score Expect Identities Positives Gaps  23.1 bits(47)52 13/25(52%) 13/25(52%) 9/25(36%)  Query 13 SGYGSPGKK------LIKKIL-KIL 30  SGY P KK LI KI KIL  Sbjct 194 SGY--PLKKEDSEATLINKIVKKIL 216 |
| 1. Alignment statistics for the chimeras CD |
| 1. N-terminal   hypothetical protein DVH24_024111 [Malus domestica]  Sequence ID: RXH94427.1  Score Expect Identities Positives Gaps  25.2 bits(52)7.8 7/10(70%) 7/10(70%) 0/10(0%)  Query 2 WQRRIRRWRR 11  W RR RR RR  Sbjct 303 WRRRRRRRRR 312   1. N-terminal plus linker   Subtilisin-like protease SBT6.1 [Malus domestica]; Sequence ID: XP_008361242.  Score Expect Identities Positives Gaps  26.9 bits(56)2.0 10/17(59%) 10/17(58%) 4/17(23%)  Query 1 FW---QRRIRRWRRGSG 14  FW Q R RR R GSG  Sbjct 1021 FWRIRQKR-RRRRKGSG 1036   1. The whole length   late embryogenesis abundant protein M17-like [Malus domestica]  Sequence ID: XP_028963845.  Score Expect Identities Positives Gaps  35.0 bits(75)0.003 16/36(44%) 16/36(44%) 15/36(41%)  Query 2 WQR------R-IR-RWRRGSGYGSPGRR--WWRWWR 27  WQR R R RW RG GRR WRW R  Sbjct 160 WQRWRGQGRRGTRWRWKRG-----QGRRGTRWRWRR 190   1. Linker plus C-Terminal   uncharacterized protein LOC103424581 [Malus domestica]; Sequence ID: XP_008360894.  Score Expect Identities Positives Gaps  24.4 bits(50)15 8/16(50%) 9/16(56%) 1/16(6%)  Query 11 RGSGYGSPGRRWWRWW 26  R S + GRR W WW  Sbjct 123 RASSFSKHGRR-WSWW 137 |
| 1. Alignment statistics for the chimeras DC |
| 1. N-terminal   protein DETOXIFICATION 19 [Citrus sinensis]; Sequence ID: KAH9802589.1  Score Expect Identities Positives Gaps  28.2 bits(59)3.7 7 /8(88%) 7/8(87%) 0/8(0%)  Query 1 RRWWRWWR 8  RRWWR WR  Sbjct 33 RRWWRKWR 40   1. Linker plus C-terminal   chaperone protein ClpB1 [Citrus sinensis]; Sequence ID: XP_006477653.1  Score Expect Identities Positives Gaps  26.9 bits(56)10 9/19(47%) 10/19(52%) 5/19(26%)  Query 10 SYGSVYGSPGFWQRRIRRW 28  SY +YG R IRRW  Sbjct 809 SYDPIYGA-----RPIRRW 822 |
| 1. Alignment statistics for the S-S bridged L19GA |
| 1. N-terminal   aspartyl protease family protein 2-like [Malus domestica]; Sequence ID: XP_008343434.2  Score Expect Identities Positives Gaps  24.8 bits(51) 4.4 7/8(88%) 7/8(87%) 1/8(12%)  Query 2 CRRLCYKQ 9  CRR CYKQ  Sbjct 175 CRR-CYKQ 181 |
| 1. C-terminal   peamaclein [Malus domestica]; Sequence ID: XP_008360977.1  Score Expect Identities Positives Gaps  24.8 bits(51) 4.2 6/9(67%) 7/9(77%) 0/9(0%)  Query 7 YKQRCVTYC 15  YK+RC YC  Sbjct 43 YKERCLKYC 51 |
| 1. 16 out of 19   probable plastid-lipid-associated protein 13, chloroplastic isoform X2 [Malus domestica]  Sequence ID: XP_008392074.2  Score Expect Identities Positives Gaps  24.4 bits(50) 6.2 10/21(48%) 10/21(47%) 8/21(38%)  Query 2 CRRLCYK------QRCVTYCR 16  CRR CY QR V CR  Sbjct 46 CRRKCYRDGRISFQRSV--CR 64 |

**Table sIIa.** *C*Las clearance by different chimeras by detached leaf assays

**Table sIIb.** *E. amylovora* clearance by different chimeras by detached leaf assays
